# The aorta in humans and African great apes, and cardiac output and metabolic levels in human evolution

**DOI:** 10.1038/s41598-023-33675-1

**Published:** 2023-04-26

**Authors:** Luis Ríos, Meg M. Sleeper, Marietta D. Danforth, Hayley Weston Murphy, Ilana Kutinsky, Antonio Rosas, Markus Bastir, José Gómez-Cambronero, Ricardo Sanjurjo, Laurence Campens, Oliver Rider, Francisco Pastor

**Affiliations:** 1 Unit of Physical Anthropology, Department of Biodiversity, Ecology and Evolution, Faculty of Biological Sciences, Universidad Complutense de Madrid, 28040 Madrid, Spain; 2 Department of Physical Anthropology, Aranzadi Sciences Society, 20014 Donostia, Basque Country Spain; 3 Paleoanthropology Group, Department of Paleobiology, Museo Nacional de Ciencias Naturales (MNCN-CSIC), 28006 Madrid, Spain; 4 Department of Small Animal Clinical Sciences, College of Veterinary Medicine, University of Florida, 2015 SW 16th Avenue, PO Box 100126, Gainesville, FL 32610-0126 USA; 5 Great Ape Heart Project, Detroit Zoological Society, 8450 W. 10 Mile Rd., Royal Oak, MI 48067 USA; 6 Oakland University William Beaumont School of Medicine, 586 Pioneer Drive, Rochester, MI 48309 USA; 7 Cardiology Department, Ghent University Hospital, 9000 Ghent, Belgium; 8 University of Oxford Centre for Cardiac Magnetic Resonance Research, Division of Cardiovascular Medicine, Radcliffe Department of Medicine, University of Oxford, John Radcliffe Hospital, Oxford, OX3 9DU UK; 9 Department of Anatomy and Radiology, University of Valladolid, 47005 Valladolid, Spain

**Keywords:** Anthropology, Animal physiology

## Abstract

Humans have a larger energy budget than great apes, allowing the combination of the metabolically expensive traits that define our life history. This budget is ultimately related to the cardiac output, the product of the blood pumped from the ventricle and the number of heart beats per minute, a measure of the blood available for the whole organism physiological activity. To show the relationship between cardiac output and energy expenditure in hominid evolution, we study a surrogate measure of cardiac output, the aortic root diameter, in humans and great apes. When compared to gorillas and chimpanzees, humans present an increased body mass adjusted aortic root diameter. We also use data from the literature to show that over the human lifespan, cardiac output and total energy expenditure follow almost identical trajectories, with a marked increase during the period of brain growth, and a plateau during most of the adult life. The limited variation of adjusted cardiac output with sex, age and physical activity supports the compensation model of energy expenditure in humans. Finally, we present a first study of cardiac output in the skeleton through the study of the aortic impression in the vertebral bodies of the spine. It is absent in great apes, and present in humans and Neanderthals, large-brained hominins with an extended life cycle. An increased adjusted cardiac output, underlying higher total energy expenditure, would have been a key process in human evolution.

## Introduction

The cardiac output is the product of the amount of blood pumped from a ventricle in a single heartbeat (stroke volume), and the number of heart beats per minute (heart rate). It is a measure of the quantity of blood available for the whole organism physiological activity during its different life stages. In metabolic terms, this whole activity is measured by the total energy expenditure (TEE), and includes energy spent in basal metabolic processes for the correct functioning and maintenance of the body systems (basal energy expenditure), energy spent in physical activity, thermoregulation and digestion of food, and energy invested during growth and reproduction^1–3^. The TEE is ultimately related to the blood available to the organism or cardiac output.

From an evolutionary perspective, the study of TEE is linked to the human energetic paradox^1^, namely, the unfolding during our lifespan of metabolically expensive traits like the growth and maintenance of a larger brain, higher rate of reproduction, high levels of physical activity, and longer lifespan than any other living hominid. Several hypotheses have been proposed to explain the origin of the extra energy needed to support these human traits (trade-off between organ’s size, energetic efficiency of locomotion, biocultural reproduction, and dietary changes), including the hypothesis of the evolution in humans of an accelerated metabolic rate and thus larger energy budget^1^. Another important debate focuses on the models explaining TEE in humans, activated by the recent findings that populations with different levels of physical activity presented similar TEE values^4,5^. This debate is framed with two alternative models, additive and constrained^2^. In the former, TEE is a linear function of physical activity, while in the latter, moderate increases in physical activity lead to increases in TEE, but after reaching higher levels of physical activity, the body compensates further increases in physical activity by reducing energy spent on other physiological activities^2^. The proposed metabolic acceleration in humans, and the finding of similar TEE values with different levels of physical activity, would point to a higher and somewhat constrained energy budget for humans, the compensation or constrained model of TEE^2^.

The blood supply has been recently studied in skeletons in relation to brain size and metabolism within primate and hominin evolution^6–8^, and in relation to locomotion and physical activity in mammals, birds, and dinosaurs^9–11^. Whether related to an organ growth or maintenance, or to locomotion and physical activity, these studies focus on the distal blood supply by studying the bony canals through which arteries pass. Following recent work focused on the heart^12^, we propose to study the total blood supply or cardiac output, a measure of the blood available for the whole organism physiological activity, from evolutionary and life history perspectives as related to the debates about TEE.

From a broader evolutionary perspective, the variation of cardiac output should reflect the variation in TEE observed between humans and great apes^1^. To test this hypothesis we first compare the aortic root diameter at the Sinus of Valsalva, a surrogate measure of the cardiac output, in living humans and African great apes. Then, with data from the literature, we study in humans the relation between cardiac output with physical activity. From a life history level approach, the variation of both cardiac output and TEE should be related across the lifespan of the organism^3^, and we test this hypothesis by studying the change across the lifespan of the cardiac output, TEE, and brain weight. Finally, a potential skeletal cardiovascular marker, the aortic impression left by the descending aorta on the vertebral bodies of the spine, is studied in skeletons of modern humans and great apes, as well as in two hominin fossils (Material and Methods, and Supplementary Material 1).

## Results

**Figure 1:**
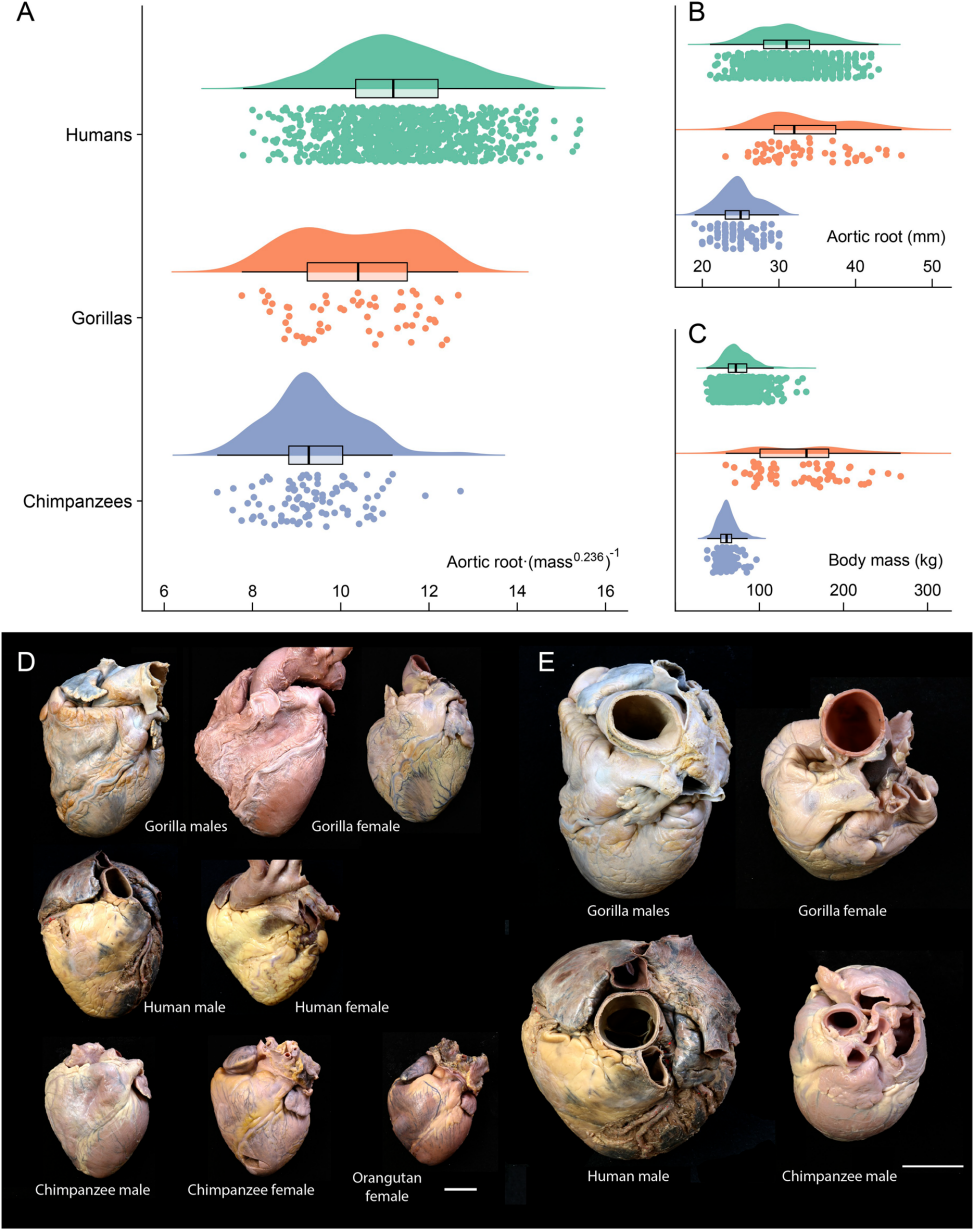
Differences between humans, gorillas, and chimpanzees (sexes combined), in aortic root diameter scaled to body mass raised to the exponent 0.236 (**A**), aortic root diameter (**B**), and body mass (**C**) (see Supplementary Materials 2–4). The raw data and probability density, together with a box plot (median and interquantile range), are shown in these raincloud plots^77^. All comparisons between species were statistically significant (*P* < 0.001), except for the aortic root diameter between humans and gorillas (*P* = 0.208). *P* values correspond to Games-Howell post hoc tests, corrected for family wise error (36 tests), with the Bonferroni-Holm method (see Supplementary Material 2). In (**D**), anterior view of hearts from anatomical dissections carried out by one of the authors (F.P.) for teaching purposes in the Faculty of Medicine (Valladolid, Spain) (Supplementary Material 3). From top to bottom and from left to right, two male and one female gorilla hearts (who died in different Zoological Parks in Spain); one male and one female human heart from donated bodies; one male and one female chimpanzee hearts together with a female orangutan heart (individuals who died in different Zoological Parks in Spain). In (**E**), view of the ascending aorta of selected hearts from (**D**), the gorilla male and female (top row), and a human and chimpanzee males (bottom row). White bars, 3 cm.

Gorillas have larger body mass and slightly, non-statistically significant, higher aortic root diameter than humans, while human body mass and aortic root diameter are larger than chimpanzees (Fig. 1, Table 1, Supplementary Materials 2–3). When sex was considered, these differences were maintained, except for body mass between human and chimpanzee females (Table 1, Supplementary Material 2). In this sample of living hominids, the aortic root diameter scales allometrically to body mass raised to the exponent 0.236 (Supplementary Material 4). Humans present a higher allometrically scaled aortic root diameter than gorillas and chimpanzees (Fig. 1, Table 1) and, when considering sex, this difference was maintained although it was non-statistically significant between human and gorilla females (Supplementary Material 2).

**Table 1:** The mean (SD) and mean difference (95% CI) and p values, for body mass (BM), aortic root diameter (ARD), and aortic root diameter scaled to body mass raised to 0.236 for the three species and considering sex.

	BM (kg)	ARD (mm)	ARD (mm)/BM^0.236^
Total sample			
Humans (947)	74.50 (16.79)	31.14 (4.11)	11.30 (1.36)
Gorillas (60)	148.45 (50.43)	33.25 (5.44)	10.31 (1.32)
Chimpanzees (96)	61.51 (10.78)	24.71 (2.49)	9.38 (0.99)
H-G	− 73.94 (− 86.8, − 61.5), p < 0.001	− 2.11 (− 3.57,0.78), p = 0.08	0.99 (0.64, 1.32), p < 0.001
H-C	12.99 (10.5, 15.4), p < 0.001	6.42 (5.89, 6.95), p < 0.001	1.92 (1.69, 2.15), p < 0.001
G-C	86.93 (74.1, 99.8), p < 0.001	8.53 (7.9, 9.99), p < 0.001	0.93 (0.53, 1.32), p < 0.001
Males			
Humans (417)	81.28 (15.76)	33.51 (3.73)	11.91 (1.27)
Gorillas (34)	185.70 (31.33)	35.59 (5.63)	10.38 (1.50)
Chimpanzees (58)	62.21 (8.11)	25.5 (2.22)	9.64 (0.87)
H − G	− 104.42 (− 115, − 94.2), p < 0.001	− 2.08 (− 3.98,0.223), p = 0.30	1.52 (1.03,2.05), p < 0.001
H − C	19.06 (16.4, 21.7), p < 0.001	8.01 (7.32, 8.67), p < 0.001	2.27 (2, 2.53), p < 0.001
G − C	123.49 (113,134), p < 0.001	10.09 (8.14,12), p < 0.001	0.74 (0.18,1.28), p = 0.12
Females			
Humans (530)	69.17 (15.62)	29.28 (3.36)	10.83 (1.24)
Gorillas (26)	99.73 (18.75)	30.19 (3.28)	10.22 (1.07)
Chimpanzees (38)	60.52 (13.73)	23.52 (2.42)	9.01 (1.05)
H − G	− 30.56 (− 38, − 23.5), p < 0.001	− 0.91 (− 2.16, 0.35), p = 0.36	0.61 (0.17, 1.01), p = 0.11
H − C	8.65 (3.98, 13), p = 0.01	5.75 (4.9, 6.54), p < 0.001	1.82 (1.45, 2.14), p < 0.001
G − C	39.20 (30.9, 47.5), p < 0.001	6.66 (5.21, 8.09), p < 0.001	1.21 (0.68, 1.72), p < 0.001

*BM* body mass, *ARD* aortic root diameter. Sample sizes vary between 1 and 6 cases for different subsamples and variables (see TABLESUPP), maximum sample sizes are shown. Statistical significance of the Games–Howell post hoc tests with Bonferroni-Holm correction (72 tests) are indicated. *P < 0.05. *H − G* humans minus gorillas, *H − C* humans minus chimpanzees, *G − C* gorillas minus chimpanzees.

**Figure 2:**
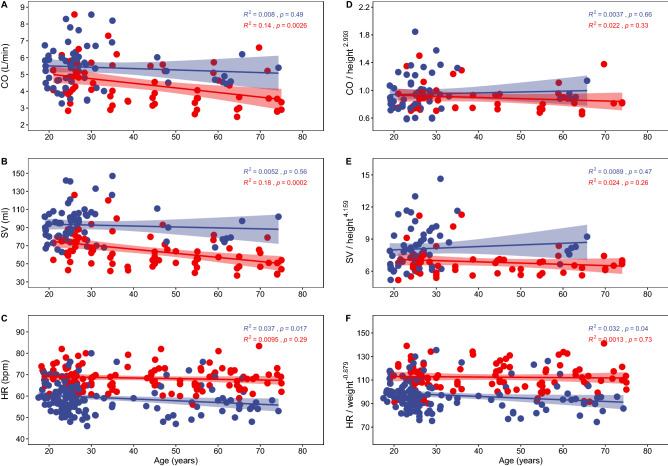
Cardiac output (CO, L/min), stroke volume (SV, mL), and heart rate (HR, beats per minute, bpm), across the adult lifespan for 101 to 277 human samples classified either as control (healthy persons who do not regularly practice sports, red dots), or physically active (either professional athletes or subjects who regularly practice sports, blue dots), obtained from a literature search (Supplementary Material 6). Values for the three variables are shown in (**A**–**C**), while values scaled to height (cardiac output, stroke volume), or weight (heart rate), raised to their correspondent exponents (see Supplementary Material 6), are shown in (**D**, **E**). Lines and shaded regions indicate simple linear regression analysis (ordinary least square, OLS), and 95% confidence intervals, with age as a predictor, and R^2^ and p values for each regression are shown (see Supplementary Material 6).

The aortic root diameter is associated with cardiac structural variables related to the left ventricle, indicating an association with stroke volume, a component of cardiac output (Supplementary Material 5). For cardiac output and its components, stroke volume and heart rate, we found a moderate or lack of decline with age in human control and athlete cross-sectional samples, especially when the variables where scaled (Fig. 2, Supplementary Material 6). No differences in the pattern of change with age (slopes) where observed for the non-scaled and scaled variables between the control and athlete samples. Significant differences between the samples (elevation) were observed for the three non-scaled variables. For the scaled variables, significant differences were observed for stroke volume and heart rate, but not for cardiac output (Fig. 2, Supplementary Material 6). When restricting the age (under 40 years), and height (minimum of 163 cm), of the control and athlete samples to make them more comparable (athlete samples were younger and taller), no significant differences were observed for the non-scaled and scaled cardiac output. This lack of difference is achieved through an inverse variation of stroke volume and heart rate, variables that present statistically significant differences between control (lower stroke volume and higher heart rate) and physically active samples (higher stroke volume and lower heart rate) (Supplementary Material 6, Supplementary Fig. 3).

**Figure 3:**
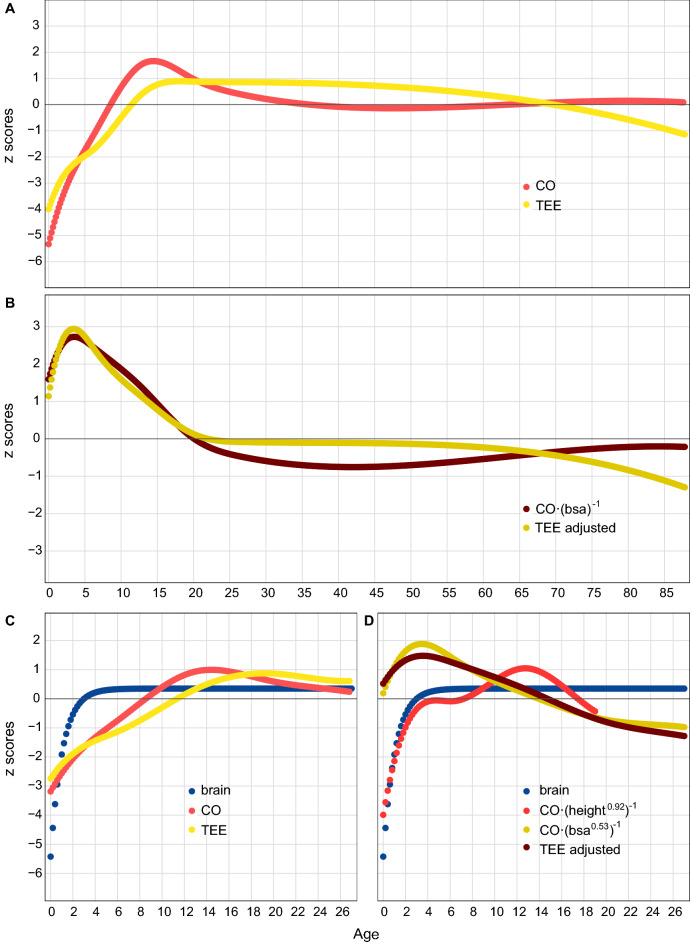
Change along the lifespan of cardiac output (CO), and total energy expenditure (TEE) (**A**), and of both variables adjusted to body surface area (CO/bsa)^13^ and fat free mass^3^, respectively (**B**). Change between birth and adulthood of brain weight, cardiac output, and total energy expenditure (**C**). In (**D**), the same variables, but with cardiac output scaled to height and body surface area^14^, and TEE adjusted to fat free mass^3^ (**B**). Data were first obtained from the literature (Supplementary Material 7), and then a Gompertz (brain), or cubic spine function (all the other variables), were fitted to the values. Predicted values from the functions were obtained and converted to z-scores for comparison between variables (Supplementary Material 7).

Regarding changes across the lifespan, absolute cardiac output increases during growth and development, and then decreases until reaching adult values during the third decade of life (Fig. 3A,C). When adjusted to two measures of body size (height and body surface area), a rapid acceleration is observed during infancy and childhood, followed either by a progressive decline (BSA adjusted)^13,14^, or another acceleration during adolescence (height adjusted), before declining to adult values (Fig. 3B,D). A similar change is observed for absolute and fat free mass adjusted TEE across the lifespan^3^ (Fig. 3A–D). During infancy and childhood, growth in brain weight follows a similar pattern (Fig. 3C,D), while growth velocity in body weight and height declines or plateau during that period of increase in brain weight and acceleration in adjusted cardiac output and TEE^15^ (Fig. 3, Supplementary Fig. 4 ,Supplementary Material 7).

**Figure 4:**
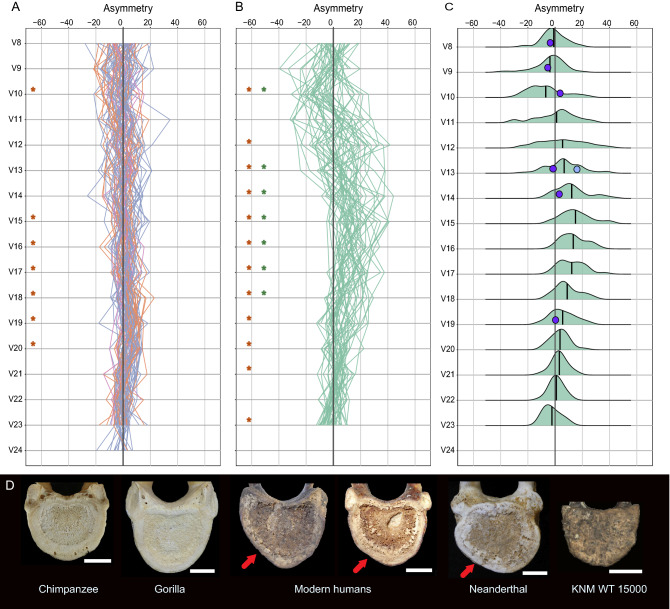
The vertebral body asymmetry values between vertebral levels V8 (T1) and V24 (see Supplementary Material 8). In A, great ape’s values are displayed (73 individuals: *Pan*, 43; *Gorilla*, 24; *Pongo*, 6), following the same color code as in Fig. 1, with orangutan specimens in pink color. In B, human values are displayed (48 individuals). Sexes were combined for great apes and humans. The red asterisk in (**A** and **B**) indicate vertebral levels where asymmetry was significant, while the green asterisks in (**B**) indicate the vertebral level where the humans were significantly more asymmetric than the great apes. In (**C**), the human density plots per vertebral level are shown, together with the mean value (black vertical line). The value from one Neanderthal from El Sidrón site is shown (light blue circle), as well as the values from the vertebrae from the KNM WT-150000 fossil (dark blue circles). In (**D**), the inferior view of the sixth thoracic vertebral body surface from one chimpanzee, one gorilla, two modern humans, one Neanderthal and the fossil KNM WT 15,000 are shown, with the white bar representing 1 cm. The red arrow in the two modern human vertebra and the Neanderthal vertebra indicates the flattening of the left side of the vertebral body left by the descending aorta, or aortic impression.

The aortic impression, described as “a variable flattening that may be found on the left side of the bodies of mid-thoracic vertebrae”^16^, was measured by the bilateral asymmetry of the anterior half of the vertebral bodies (Supplementary Material 8). The asymmetry in humans but not in great apes reflects the known course of the descending aorta along the thoracolumbar spine. At the level of T4, the aorta is located on the antero-lateral left side of the vertebral body, and throughout its descending trajectory it progressively moves towards a more anterior location. Significant asymmetry was observed continuously in vertebral levels V12 (T5) to V21 (L2) (Fig. 4, Supplementary Material 8).

## Discussion

 The aorta is the vessel through which blood passes from the heart to the rest of the body during systole. We have shown the association between aortic root diameter and cardiac structural variables of the left ventricle (Supplementary Material 5), and a significant association has been reported elsewhere between the dimensions of the aortic root and the left ventricular outflow tract, used for the estimation of stroke volume and thus for cardiac output estimation^17–19^. The aortic root diameter of gorillas is slightly larger than humans, and these findings would suggest that the cardiac output is similar in both species, despite the difference in body mass. When scaled to body mass, the aortic root diameter is larger in humans than in gorillas (and chimpanzees), in agreement with the recent observations that humans have larger stroke volume and cardiac output than chimpanzees and a small sample of gorillas^12^. A higher adjusted aortic root diameter in humans, as a surrogate measure a of a higher adjusted cardiac output, would fit the observed metabolic acceleration in humans compared with great apes in terms of total energy expenditure adjusted to fat free mass^1^. Furthermore, the observed difference in adjusted aortic root diameter could be larger if fat free mass instead of mass would have been used for scaling, since humans store more fat than great apes^1,20^. Interestingly, gorillas present a statistically significant larger adjusted aortic root than chimpanzees (Table 1), while previously reported adjusted TEE for panins is greater than that for gorillas^1^. Specifically, although the adjusted aortic root in gorillas is larger than in chimpanzees for both sexes, significance is only maintained for females (Table 1). Different factors might be involved in this discrepancy. First, the gorilla sample used for obtaining TEE data^1^ was small (10 gorillas), and biased towards females (6 females, 4 males), which might influence the results in a highly sexually dimorphic genus, with additional male variation in size associated to social rank^21,22^. Second, as mentioned above, we are using the aortic root diameter as a surrogate measure of cardiac output, which might add more variance to the extrapolation from aortic root to cardiac output, and from this to TEE. Finally, it is also important to consider that all the great ape data come from animals in captivity, and while for chimpanzees measurements were carried out by one of the authors (M.M.S.), the gorilla data were gathered from 17 different zoos from USA, implying variation due to the different technicians and devices involved in obtaining the measurements. Further studies on great ape physiology, with a special effort to include gorillas (and orangutans), are warranted.

Across the lifespan, scaled and non-scaled cardiac output at rest and its components, stroke volume and heart rate, present a moderate or lack of association with age. Regarding physical activity, it is significantly associated to variation in adjusted stroke volume and adjusted heart rate but in an inverse manner. The result is a lack of association between physical activity and adjusted cardiac output at rest. In the case of regular endurance physical activity, stroke volume increases through remodeling of the left ventricle end diastolic cavity size, accommodating greater cardiac output during exercise^23,24^. At rest, once the cavity has remodeled, the lower requirement of cardiac output at rest is maintained through a decline in resting heart rate (average of 9 beats per minute, Supplementary Material 9), probably due to a decrease in the intrinsic heart rate^25^. In fact, besides physical activity, variation in heat rate seems to be limited to sex, with women presenting a heart rate on average 3 beats per minute higher^26–28^. It is slightly or not associated to height, weight, ancestry, and age^26–28^, with small variations found in high altitude residents^29^ (Supplementary Material 9). If we consider maximal heart rate across the lifespan, it is predicted by age to a large extent, and its rate of decline is not associated with either sex or moderate levels of physical activity^30^, although under forty years of age it has been observed to be lower in athletes compared with age matched sedentary controls^31^. The immediate response and chronic adjustment to physical activity implies synergizing numerous tissues from different organ systems in a complex response that will need large scale, multi-omic studies (genomic, transcriptomic, proteomic, metabolomic) to be elucidated^23,32,33^. The result of this complex response to regular endurance physical activity allows an increase in cardiac output during exercise while maintaining its value at rest, by increasing stroke volume and decreasing heart rate.

A modification of cardiac output at rest is only observed in organismal changes such as pregnancy^34^, when several physiological parameters are altered^35^. The sexual difference in heart rate augments during pregnancy (average of 7.6 bpm)^36^, adjusting cardiac output to the increase in fat free mass, thus keeping the energy use of pregnant women scaled with their body size, as recently observed^3^. Pathological states affecting basic parameters like the oxygen supply to the tissues, or the level of a general hormone such as the thyroid hormone, also affects cardiac output at rest. In anemia, tissue hypoxia triggers compensatory non-hemodynamic and hemodynamic mechanisms, and among the latter, increased resting cardiac output is the main mechanism with low levels of hemoglobin (< 10 g/dl)^37^. The thyroid hormone, with differences between humans and great apes and within great apes^38,39^, regulates basic metabolic processes involved in growth and energy expenditure^40–42^. Cardiac output and resting energy expenditure increase in hyperthyroidism, with exercise intolerance, and decrease in hypothyroidism^41^. The moderate or lack of association of adjusted cardiac output at rest with physical activity (and with sex and age), and the fact that permanent changes are only observed in organismal changes such as pregnancy, or in pathophysiological states affecting basal parameters (hemoglobin, thyroid hormone), indicate a mechanism to restrict variation of this fundamental physiological variable.

On the other hand, in humans, the changes in absolute and adjusted cardiac output and TEE across the lifespan^3^, are similar (Fig. 3A,B), especially during the first years of postnatal life (Fig. 3C,D). For the adjusted cardiac output and TEE, this similarity is observed despite the datasets coming from different samples^3,13^, and despite TEE being adjusted to fat free mass, better reflecting the metabolically active tissue. The increase in both variables during the first years of postnatal life coincides with the period of brain growth and development (Fig. 3C,D), a metabolically costly process that uses above 40% of the body’s daily energy requirement, and over 66% of the body’s resting metabolism, requiring a trade-off with body growth^15^ (Supplementary Fig. 4). The relation between brain volume and total cerebral blood flow in children under 7 years of age and in adults^43,44^, points to the association between early brain growth and the early increase in cardiac output. From 20 to 60 years, absolute and adjusted cardiac output and TEE reach a plateau (see also Fig. 2), in agreement with previous works that show a slight or lack of decline of cardiac output with age^45,46^.

The human accelerated metabolism possibly evolved in a context of regular, moderate, endurance physical activity across the lifespan ^47^, which resulted in structural changes in the heart optimizing cardiac output for endurance physical activity^12,24^. This metabolic acceleration would accommodate the energetic costs of modern human life history^1^, like growing a large brain (Fig. 3) and adjusting to the wide range of human physical activity (Fig. 2), which is associated to important consequences for health^12,23–25,32,33,48^. As summarized in the introduction, two models frame the debate about TEE in modern humans, additive and constrained^2^. The constrained model was supported by the finding that populations^4,5^, or individuals^2^, with different levels of physical activity, presented similar TEE values, including children^49^. More in detail, there are three models of energy management, namely, additive, performance, and compensation (constrained). Each model has its own testable set of predictions regarding the relation between TEE, basal energy expenditure and physical activity^50^, and further evidence has been found supporting the constrained or compensation model under conditions of physical activity^51,52^. The restricted variation of cardiac output at rest, and its parallel change across the lifespan with TEE, with a plateau during most of the adult life, would lend support to the compensation model of TEE.

Turning to the fossil record, in primate and human evolution the blood supply has been recently studied in skeletons in relation to brain size and metabolism^6–8^. These studies focus on the distal blood supply by studying the bony canals through which arteries pass, and an increase in the total cerebral blood flow rate has been observed in hominin evolution. In this work, we have focused on the cardiac output, or total blood supply of the organism. As an initial approximation to its study in skeletal remains, we have studied the aortic impression through the anterior vertebral body asymmetry. This measure captures more asymmetry than that strictly related to the aortic impression (Supplementary Material 8), and as an additional check, each vertebra was visually assessed to detect any asymmetry caused by a unilateral flattening on the left side of the body. Besides, the trajectory of asymmetry along the vertebral column within individuals is not smooth, and where a complete vertebral column is lacking, absence of an aortic impression in an isolated vertebra should be considered with caution. With these considerations in mind, we observed that the aortic impression is present in the human spine and absent in the great ape spine. Regarding fossil hominins, a Neanderthal vertebra presents a clear aortic impression, while it is absent in the KNM WT 15,000 vertebrae.

Gorillas and humans present a large aorta, but with different body mass and thorax size and shape. A descending aorta similar in size would be placed within a smaller and flatter ribcage in humans, and within a larger and deeper ribcage in gorillas^53^, a fact that could imply a tighter spatial packing of the ribcage content in humans, thereby contributing to the development of the aortic impression in humans. Neanderthals present a slightly deeper thorax than modern humans^54^, but the vertebra from El Sidrón presents a clear aortic impression. Neanderthals and modern humans are large brained hominins with a similar growth pattern and an extended life cycle^55^, energetically expensive traits. The metabolic acceleration required to sustain these traits could be provided by a larger cardiac output by body mass unit. Thus, an hypothetical explanation for the aortic impression is that it is an skeletal signal of a large aorta, within a reduced ribcage, in hominins with a combination of energetically expensive traits^55^. In this scenario, KNM WT 15,000, without an aortic impression, presents a smaller brain and absence of the features of the modern human growth pattern^56^, a combination of features energetically less expensive than those present in humans and Neanderthals. This would point to a smaller aorta and lower adjusted cardiac output. On the other hand, the capability of endurance running has been widely discussed for this hominin^47^, including the recent reconstruction of its thorax, with an unexpected shape^57^, considerably deeper antero-posteriorly than in modern humans, a configuration that could even be unrelated to the evolution of endurance running performance^58^. The claim of endurance running would point to an increased metabolic activity and, like gorillas, it might be possible that a large aorta in a deep thorax could leave no aortic impression, although the thorax and body mass of gorillas and KNM WT 15,000 are different. The expensive traits central to the human energetic paradox would have been fueled by an increase in TEE or metabolic acceleration, which in turn would be related to an increase in the adjusted cardiac output of the organism. Further physiological and skeletal studies will be needed to test the hypothesis that the aortic impression, absent in KNM WT 15,000 and present in Neanderthals and modern humans, is an osteological signal of an increased adjusted cardiac output in hominin evolution.

## Conclusions

We study the total blood supply or cardiac output of the organism, from evolutionary and life history perspectives as related to the debates about total energy expenditure in humans. Humans present a higher, body mass adjusted, aortic root diameter than gorillas and chimpanzees. As a surrogate measure of the cardiac output, this would imply a higher adjusted cardiac output in humans than in great apes, in agreement with the human metabolic acceleration in terms of total energy expenditure adjusted to fat free mass. In humans, body mass adjusted cardiac output at rest is a fundamental physiological variable with a restricted variation regarding sex, age, and physical activity. Furthermore, the patterns of change in absolute and adjusted cardiac output and total energy expenditure across the lifespan are similar, with a marked increase in both variables during the first years of postnatal life, coincident with the period of brain growth and development, and with a plateau during most of the adult life. In the debate about models explaining total energy expenditure in humans, this pattern of variation of cardiac output would lend support to the compensation model. A first approximation of the study of the organismal total blood supply in the primate fossil record is presented through the study of the aortic impression in the vertebral bodies of the spine. It is present in humans and Neanderthals, large-brained hominins with an extended life cycle. The aortic impression could be a skeletal signal of a large aorta, within a reduced ribcage, in hominins with a combination of energetically expensive traits requiring a metabolic acceleration. The underlying increase in the adjusted cardiac output of the organism would have been a key process in human evolution.

## Methods

The human sample come from two studies carried out at Ghent University Hospital (Belgium), and at the University Oxford Centre for Clinical Magnetic Resonance Research (OCMR). The description of the study cohorts, and the methods followed, haven been described and published previously^59,60^ and are summarized here. Both studies were approved by the Ethical Committee of the University Hospital Ghent, and Oxfordshire Research Ethics Committee, respectively, and all methods were carried out in accordance with relevant guidelines and regulations. Subjects were screened for the presence of identifiable diseases and excluded for the analysis. Only adult subjects > 18 years of age were included (see Supplementary Material 1). Height and weight (body mass) were recorded for every subject. Based on these criteria, a total of 530 females (mean age 46.7 years), and 417 males (mean age 46.8 years) were included in the study. For the echocardiographic sample, transthoracic echocardiography was performed by experienced adult and pediatric cardiologists using commercially available ultrasound equipment (Vivid 7; GE Vingmed Ultrasound AS, Horten, Norway) with adequate multifrequency transducers, ranging from 3.5 to 8 MHz for children and from 2 to 5 MHz for adults. All proximal aortic diameters were assessed on 2-dimensional images in the parasternal long-axis view at end-diastole from leading edge to leading edge^61^. All aortic measurements were performed off-line by three experienced readers (including one of the present authors, L.C.). The aortic root diameter at the sinuses of Valsalva was selected for study. For the cardiovascular magnetic resonance sample, Imaging was performed on a 1.5 Tesla MR system (Siemens Avanto, Erlangen, Germany). All imaging was retrospectively cardiac gated with a precordial three lead ECG and acquired during end expiration breath hold. Oblique sagittal pilot, half-Fourier single shot turbo spin echo (HASTE) images were followed by steady-state free precession (SSFP) cine images with the following parameters: echo time of 1.12 ms, repetition time of 39 ms using 15 segments and 25 phases, slice thickness of 7 mm and pixel size of 2 mm × 2 mm. Cross sectional images of the aorta were obtained orthogonal to the sagittal oblique scout^62^. A sagittal oblique SSFP view of the left ventricular outflow tract (LVOT) was acquired, allowing the aortic root diameter at the sinuses of Valsalva to be measured. Maximum diastolic diameter measurements were performed by a single reader with 8 years of CMR experience, from luminal edge to luminal edge.

The great ape sample come from two studies, and the description of the study cohorts and the methods followed, haven been previously described and published^63–65^, and are summarized here. For the chimpanzees, Echocardiographic evaluations were performed in a colony of chimpanzees between 2002 and 2011. Echocardiography was performed with a 2.5-MHz transducer and an ECG for cardiac cycle timing. Echocardiographic evaluations were performed with the animal positioned in left lateral recumbency and the left arm raised above the left shoulder. The aortic root diameter and left atrial diameter in the short and long axis during diastole were measured during 2-D echocardiography at the parasternal window. Measurements in both axes were performed in the first diastolic frame in which closure of the aortic valve was evident. For the gorillas, North American zoological institutions were invited to participate in a population-based cohort study examining cardiovascular data from captive gorillas, and data are presented from 17 institutions. Animals were placed in either left lateral recumbency or dorsal recumbency, and all examinations were performed with transthoracic echocardiography using a MHz probe, from which the aortic root diameter was obtained. (Supplementary file 1).

For the aortic root, body mass and aortic root scaled to body mass, assumptions of normality and equality of variances were not met by at least half of the variables for the different samples (by species, and by species and sex) (assessed by density plots, Shapiro–Wilk test, p value < 0.05). Samples sizes were clearly unbalanced, with a human sample tenfold larger than the gorilla and chimpanzee samples, similar in size. Statistical significance was tested with the Games–Howell test and the Bonferroni-Holm correction was applied, reporting the adjusted p values (Supplementary Material 2). Statistical log transformations of aortic root diameter and body mass, and linear regression (logARD = b × logBM + logb) were used to determine the scaling exponent for body mass (Supplementary Material 4).

A literature search was undertaken to find articles containing basic anthropometric data (sex, age, height, body mass), diameter of the aortic root at the sinus of Valsalva, cardiac output, stroke volume, heart rate, and any of the following cardiac structural variables: left ventricle end diastole diameter (LVEDD), left ventricle end systole diameter (LVESD), left ventricle end diastole volume (LVEDV), left ventricle end systole volume (LVESV), and left ventricle mass (LVMASS). When not present, and if possible, stroke volume and cardiac output were calculated first from LEDV and LESV, and then from stroke volume and heart rate respectively (Supplementary Materials 5 and 6, Supplementary Files 2–5; references in Supplementary File 6). Samples were classified either as control (healthy subjects who do not regularly practice sports), or athlete (either professional athletes or subjects who regularly practice a specific sport). Ordinary least square (OLS) regression was selected to study the association between these variables, although the data were not individual cases but mean values from study samples. The criteria for the choice of the regression technique have been discussed elsewhere, between OLS and reduced major axis (RMA). Based on these considerations, we decided to use OLS (Supplementary Material 5 and 6).

A literature search was undertaken to find articles containing data on cardiac output, organ weight, height and weight, and total energy expenditure (TEE), across the lifespan or along the growth period. For cardiac output, results from research conducted on a local, healthy sample from Hong Kong, aged 0–60 years were selected^13,66–68^. For organ weight, results from a cadaver study on a nationwide Japanese sample were selected^69^. For height and weight, for illustrative purposes, and due to its completeness, data from the CDC Growth Charts were also selected^70^. For TEE, results obtained through the doubly labeled water method were selected, obtained in subjects aged 8–95 years^3^. (Supplementary Files 7–12). A literature search was undertaken to find articles containing data on cardiac output, organ weight, height and weight, and TEE, across the lifespan or along the growth period. Basic data were extracted from the article’s tables and figures. In order to compare data from different variables, we followed a procedure described previously^15^ (Supplementary Material 7). Continuous functions were fit separately to all the variables and different models were evaluated for the age range 0–19 or 0–27 years. Once the models were selected, predicted values were calculated at 0.2 years intervals for all the variables. Z-scores were then calculated from those predicted variables (Supplementary Material 7).

Regarding the human and great ape hearts shown in Fig. 1, they were not obtained from the present study, they were already curated at the Faculty of Medical Sciences, Universidad de Valladolid (Spain), for teaching and research purposes. The two human hearts come from anatomical dissections of cadavers donated to the Body Donation Program, Universidad de Valladolid, which includes an informed consent and two witnesses to the donation. Regarding the great ape hearts, cadavers of great apes that die in Spanish zoos are frequently send to the Faculty of Medical Sciences, Universidad de Valladolid, where one of the authors carries out anatomical dissections^71–73^, preserves soft tissue samples, and skeletonizes the cadavers.

Regarding the skeletal sample, great ape specimens came from the Africa Museum (Tervuren, Belgium), Museo Anatómico (Faculty of Medical Sciences, Universidad de Valladolid, Spain), Estación Biológica de Doñana (EBD-CSIC, Sevilla, Spain), and Museu de Ciències Naturals (Barcelona, Spain). Modern human skeletons belong to the Luís Lopes Anthropological Collection, MUHNAC (Lisbon, Portugal). Two additional fossil specimens were included, a Neanderthal vertebra from El Sidrón (Asturias, Spain), curated by one of the authors (A.R.) at the Museo Nacional de Ciencias Naturales (MNCN-CSIC, Madrid, Spain), and images from the vertebrae of KNM WT 15,000, from 3D surface scans obtained from the original fossil by other author (M.B.) (Nairobi, National Museums of Kenya). Complete, non-pathological, preferably young adult spines were selected for study. Completeness and correct sequence of the vertebrae from T1 to L5 were checked anatomically. Photographs were taken for each inferior endplate with a Canon EOS 70D with ef-s 18–135 mm f/3.5–5.6 lenses, with the endplate surface parallel to the lens of the camera. The image was transferred to Adobe Photoshop (CS6) to obtain quadrants of the endplate surface, and the surface area of the anterior left/right quadrants were measured with ImageJ^74^. Asymmetry was calculated for each vertebra following a formula for relative asymmetry that standardizes to within-individual percentages, with positive values indicating a larger right side^75^. Parametric (one sample t-test, independent samples t-test) tests were applied for detecting significant asymmetry in each vertebra, and genus-based differences in asymmetry. (Supplementary Material 8, Supplementary Files 13, 14). All graphs were developed with R^76^ (code available in Supplementary File 14).

## Supplementary Information

Supplementary Information 1.

Supplementary Information 2.

Supplementary Table 1.

Supplementary Table 2.

Supplementary Table 3.

Supplementary Table 4.

Supplementary Table 5.

Supplementary Information 3.

Supplementary Table 6.

Supplementary Table 7.

Supplementary Table 8.

Supplementary Table 9.

Supplementary Table 10.

Supplementary Table 11.

Supplementary Table 12.

Supplementary Table 13.

## Data Availability

Most data generated or analyzed during this study are included in the Supplementary Information files. The rest of the data are available from the corresponding author on reasonable request.
