# Supplementary material for: The aorta in humans and African great apes, and cardiac output and metabolic levels in human evolution
Source: Sci Rep. 2023 Apr 26;13:6841. doi: 10.1038/s41598-023-33675-1 (PMC10133235; doi:10.1038/s41598-023-33675-1)
Supplement: Supplementary file 1 — Supplementary Information 1. [file 41598_2023_33675_MOESM1_ESM.pdf]

SUPPLEMENTARY MATERIAL

THE AORTA IN HUMANS AND AFRICAN GREAT APES, AND CARDIAC OUTPUT AND METABOLIC LEVELS IN HUMAN EVOLUTION

Luis Ríos[1,2,3]*, Meg M Sleeper[4], Marietta D. Danforth[5], Hayley Weston Murphy[5], Ilana Kutinsky[6], Antonio Rosas[3], Markus Bastir[3], José Gómez-Cambronero[1], Ricardo Sanjurjo[1], Laurence Campens[7], Oliver Rider[8], Francisco Pastor[9]

1 Unit of Physical Anthropology, Department of Biodiversity, Ecology and Evolution, Faculty of Biological Sciences, Universidad Complutense de Madrid, 28040 Madrid, Spain

2 Department of Physical Anthropology, Aranzadi Sciences Society, 20014 Donostia, Basque Country, Spain

3 Paleoanthropology Group, Department of Paleobiology, Museo Nacional de Ciencias Naturales (MNCN-CSIC), 28006 Madrid, Spain

4 Department of Small Animal Clinical Sciences, College of Veterinary Medicine, University of Florida 2015 SW 16th Avenue, PO Box 100126, Gainesville, FLorida 32610-0126, USA

5 Great Ape Heart Project, Detroit Zoological Society, 8450 W. 10 Mile Rd., Royal Oak, MI 48067, USA

6 Oakland University William Beaumont School of Medicine 586 Pioneer Drive, Rochester, MI 48309, USA

7 Cardiology Department, Ghent University Hospital, Ghent 9000, Belgium

8 University of Oxford Centre for Cardiac Magnetic Resonance Research, Division of Cardiovascular Medicine, Radcliffe Department of Medicine, University of Oxford, John Radcliffe Hospital, Oxford OX3 9DU, UK

9 Department of Anatomy and Radiology, University of Valladolid, 47005 Valladolid, Spain

SUPPLEMENTARY MATERIAL 1 3

SUPPLEMENTARY MATERIAL 2 4

SUPPLEMENTARY MATERIAL 3 9

SUPPLEMENTARY MATERIAL 4 10

SUPPLEMENTARY MATERIAL 5 12

SUPPLEMENTARY MATERIAL 6 16

SUPPLEMENTARY MATERIAL 7 22

SUPPLEMENTARY MATERIAL 8 24

SUPPLEMENTARY MATERIAL 9 27

SUPPLEMENTARY MATERIAL 1

Only adult individuals were studied from the echocardiographic and NMR samples. Age at linear growth or body mass completion, or near completion, was used as the criterion for adulthood. The age at attainment of adult height is variable between human populations [1], but considering that the human samples come from modern European populations (UK and Belgium), an approximate age of 18 years for completion or near completion of adult height in both males and females was considered. For gorillas and chimpanzees, we follow the available data from the literature, bearing in mind that the age of attainment of adult dimensions is also variable between species and populations within those genera, for instance between wild and captive populations [2,3]. For gorillas, we followed Galbany et al. (2017) [4], who indicate that in their sample of wild mountain gorillas, 98% of maximum body length, back width and arm length were reached by females at 11.7, 11.9 and 15.9 years old, and by males at 13.1, 15.7, 14.5 years old respectively. Thus, as an average from these ages, we selected those individuals older than 13 years (females), and 14 years (males) as adults. For chimpanzees, Hamada and Udono (2002) [5] observed that the age of body length maturation does not differ by sex, and it occurs approximately at 12 years. Pusey et al. (2005) [6] data from Gombe show that growth in body mass slowed at 10 years of age for females and 13 years for males, while Machanda et al. (2015) [7] data from Uganda show that for males linear dimensions reached adult values by 10 years old while body areas reached adult values between the ages of 15-17 years. Thus, as an average from these ages, we selected those individuals older than 12 years old. In humans it has been observed that the diameter of the aortic root increases with age throughout adult life (see below), and it would be reasonable to expect an increase with age in other hominoids. But we decided not to put an upper limit in the age of the samples from the three species due to potential comparability problems for the length and equivalence of the adult period in the three species.

SUPPLEMENTARY MATERIAL 2

For the echocardiographic and magnetic resonance sample, assumptions of normality and equality of variances were assessed by density plots and the Shapiro-Wilk test and through the Brown-Forsythe test respectively (P value <0.05). These assumptions were not met by at least half of the variables for the different samples (by species, and by species and sex). In addition, the samples sizes were clearly unbalanced, with a human sample tenfold larger than the gorilla and chimpanzee samples, similar in size (Supplementary Table 1). From the above information we chose to test statistical significance with the Games-Howell test and, since this study was planned as hypothesis testing research and we wanted to avoid a type I error, we applied the Bonferroni-Holm correction [8], reporting the adjusted P values. The number of tests for this correction was 36, since we considered nine subsamples (species, male, female) and four variables (ARD and BM, and the two exponents of BM that appropriately normalized ARD: $ARD/BM^{0.236}$, $ARD/BM^{0.25}$). For exploratory purposes, Welch unpaired tests, and Kruskall-Wallis test with Dunn's post hoc tests, both with Bonferroni-Holm corrections, were also computed and compared against the results from the Games-Howell test. The results from the three tests were basically similar (results not shown). In Supplementary Table 1 we can observe the mean, SD, and statistical significance for BM, ARD, $ARD/BM^{0.236}$ and $ARD/BM^{0.25}$ between species and by sex. In Supplementary Figure 1, we can observe the mean difference (95% CI) for BM, ARD and $ARD/BM^{0.236}$ between species and by sex [9].

Supplementary table 1. Mean (SD) and mean difference (95% CI) for body mass, aortic root diameter, and aortic root diameter scaled to $BM^{0.236}$ and $BM^{0.25}$.

**Species**	**n**	**BM (kg)**	**n**	**ARD (mm)**	**n**	**ARD / $BM^{0.236}$**	**ARD / $BM^{0.25}$**
Humans	947	74.50 (16.79	945	31.14 (4.11)	945	11.30 (1.36)	10.65 (1.28)
Gorillas	60	148.45 (50.43)	60	33.25 (5.44)	60	10.31 (1.32)	9.62 (1.23)
Chimpanzees	91	61.51 (10.78)	96	24.71 (2.49)	90	9.38 (0.99)	8.86 (0.94)
H-G		-73.94 (-86.8,-61.5),p<0.001		-2.11 (-3.57,0.78),p=0.08		0.99 (0.64,1.32), <0.001	1.02 (0.698,1.33),p<0.001
H-C		12.99 (10.5,15.4),p<0.001		6.42 (5.89,6.95),p<0.001		1.92 (1.69,2.15), p<0.001	1.78 (1.57,2),p<0.001
G-C		86.93 (74.1,99.8),p<0.001		8.53 (7.9,9.99), p<0.001		0.93 (0.533,1.32), p<0.001	0.76 (0.392,1.13),p<0.001
**Males**	**n**	**BM (kg)**	**n**	**ARD (mm)**	**n**	**ARD (mm)/$BM^{0.236}$**	**ARD / $BM^{0.25}$**
Humans	417	81.28 (15.76)	416	33.51 (3.73)	416	11.91 (1.27)	9.10 (0.83)
Gorillas	34	185.70 (31.33)	34	35.59 (5.63)	34	10.38 (1.50)	9.65 (1.39)
Chimpanzees	53	62.21 (8.11)	58	25.5 (2.22)	53	9.64 (0.87)	11.20 (1.19)
H-G		-104.42 (-115,-94.2),p<0.001		-2.08 (-3.98,0.223), p=0.30		1.52 (1.03,2.05), p<0.001	1.54 (1.09,2.03),p<0.001
H-C		19.06 (16.4,21.7),p<0.001		8.01 (7.32,8.67), p<0.001		2.27 (2,2.53), p<0.001	2.10 (1.84,2.35),p<0.001
G-C		123.49 (113,134),p<0.001		10.09 (8.14,12), p<0.001		0.74 (0.179,1.28), p=0.12	0.55 (0.028,1.06),p=0.30
**Females**	**N**	**BM (kg)**	**n**	**ARD (mm)**	**N**	**ARD (mm)/$BM^{0.236}$**	**ARD / $BM^{0.25}$**
Humans	530	69.17 (15.62)	529	29.28 (3.36)	529	10.83 (1.24)	8.51 (1.01)
Gorillas	26	99.73 (18.75)	26	30.19 (3.28)	26	10.22 (1.07)	9.59 (1.00)
Chimpanzees	38	60.52 (13.73)	38	23.52 (2.42)	37	9.01 (1.05)	10.21 (1.18)
H-G		-30.56 (-38,-23.5),p<0.001		-0.91 (-2.16,0.35), p=0.36		0.61 (0.172,1.01), p=0.11	0.62 (0.211,1.01),p=0.08
H-C		8.65 (3.98,13), p=0.01		5.75 (4.9,6.54), p<0.001		1.82 (1.45,2.14), p<0.001	1.69 (1.34,2.01),p<0.001
G-C		39.20 (30.9,47.5),p<0.001		6.66 (5.21,8.09), p<0.001		1.21 (0.685,1.72), p<0.001	1.07 (0.574,1.57),p=0.003

BM, body mass; ARD, aortic root diameter. Statistical signficance of the Games-Howell post hoc tests with Bonferroni-Holm correction (36 tests) are indicated. *p<0.05. H-G, humans minus gorillas; H-C, humans minus chimpanzees; G-C, gorillas minus chimpanzees.

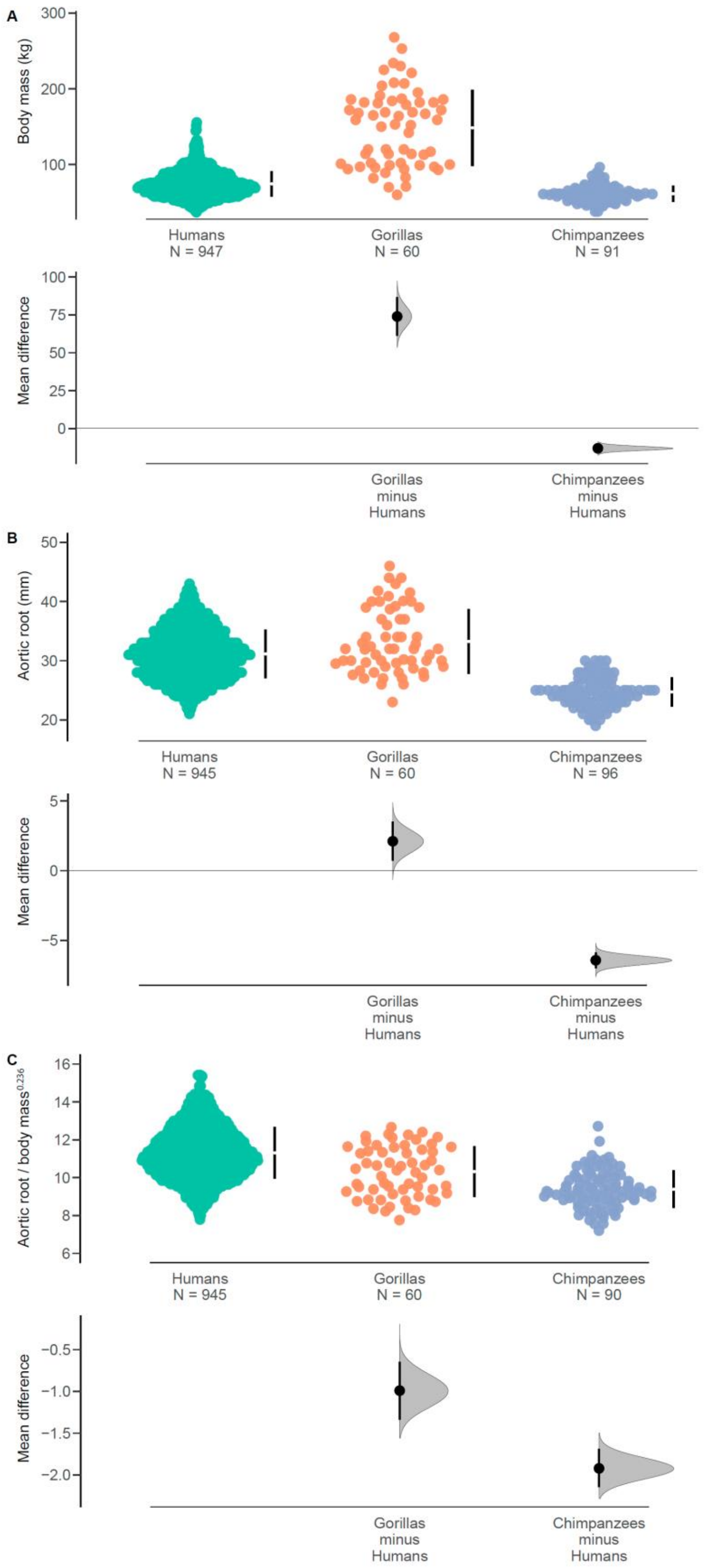

Supplementary Figure 1. Mean difference in body mass, aortic root, and aortic root/body mass$^{0.236}$, between humans and gorillas, and human and chimpanzees, for the total sample (A-C), males (D-F) and females (G-I). The raw data is plotted on the upper axes; each mean difference is plotted on the lower axes as a bootstrap sampling distribution [9]. Statistics are shown in Supplementary Table 1.

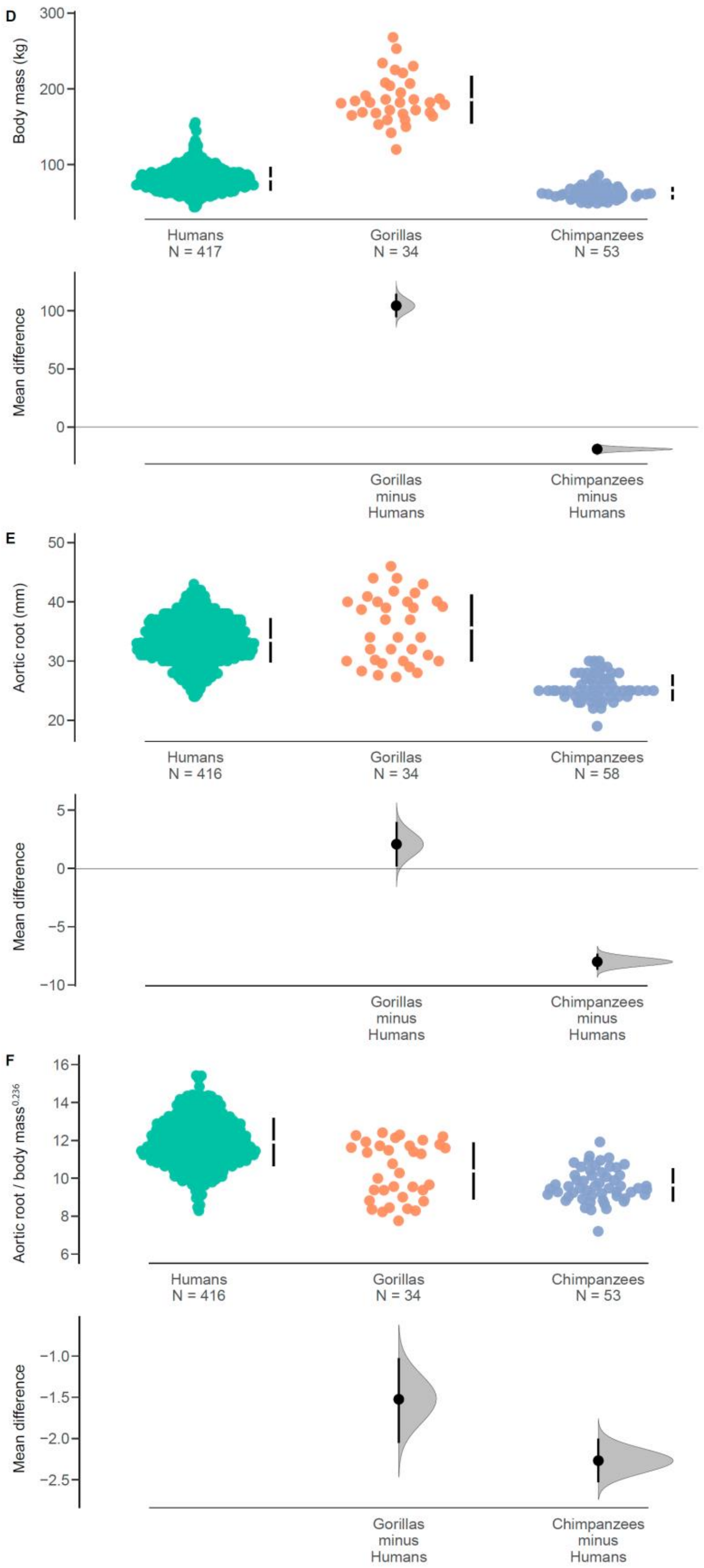

Supplementary Figure 1 (cont). Mean difference in body mass, aortic root, and aortic root/body mass$^{0.236}$, between humans and gorillas, and human and chimpanzees, for the total sample (A-C), males (D-F) and females (G-I). The raw data is plotted on the upper axes; each mean difference is plotted on the lower axes as a bootstrap sampling distribution [9]. Statistics are shown in Supplementary Table 1.

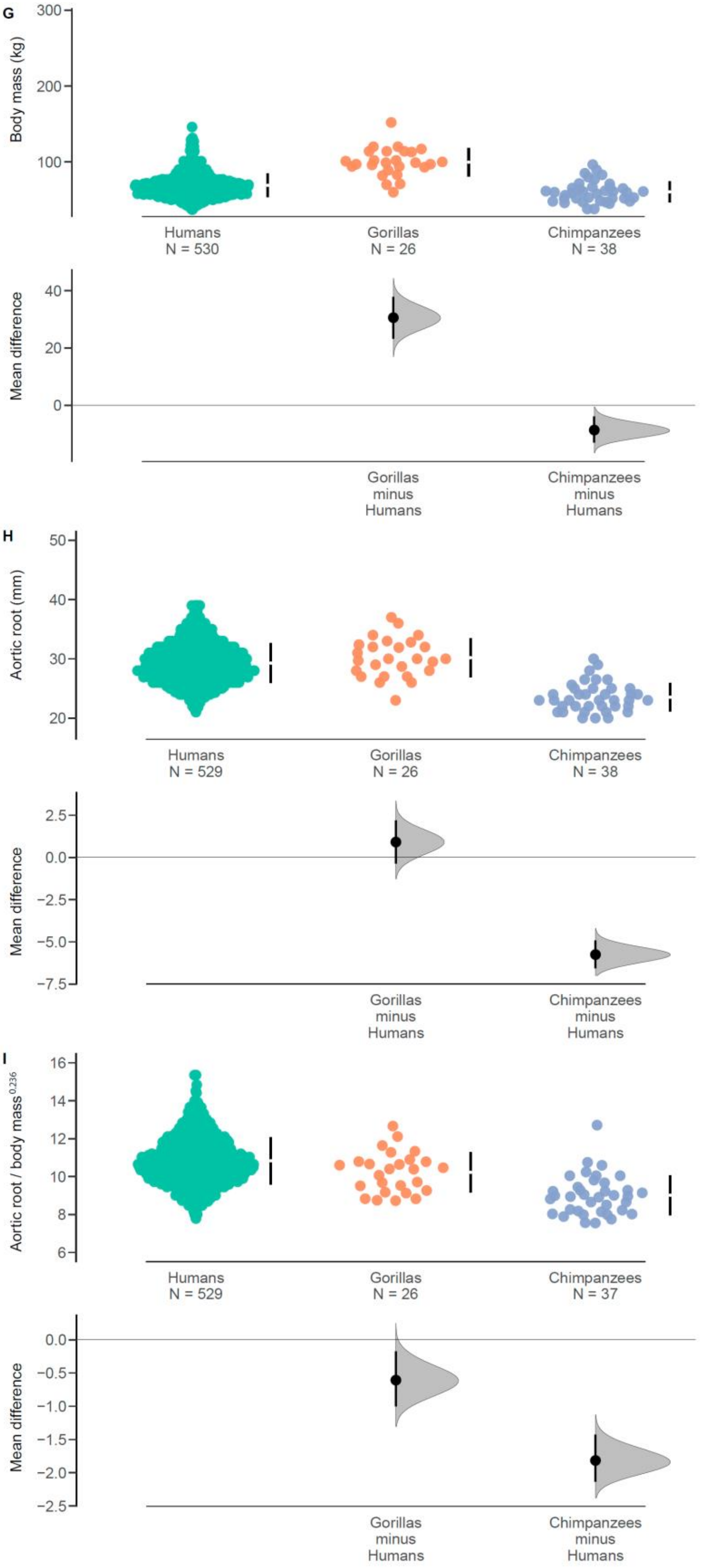

Supplementary Figure 1 (cont). Mean difference in body mass, aortic root, and aortic root/body mass$^{0.236}$, between humans and gorillas, and human and chimpanzees, for the total sample (A-C), males (D-F) and females (G-I). The raw data is plotted on the upper axes; each mean difference is plotted on the lower axes as a bootstrap sampling distribution [9]. Statistics are shown in Supplementary Table 1.

SUPPLEMENTARY MATERIAL 3

Hearts from dissection

Basic data from the human, gorilla, chimpanzee, and orangutan hearts shown in Figure 1 in the main text are presented in Supplementary Table 2. Hearts were preserved in ethanol; thus the measurements are underestimations of the true values due to tissue shrinkage.

Supplementary Table 2. Height: from the aortic root to the apex on the sternocostal surface. Perimeter: at the coronary sulcus. Aortic diameter: at the preserved ascending aorta.

ID	Species	Sex	Age	Weight (kg)	Height (cm)	Perimeter (cm)	ARD (cm)
5792	*G. gorilla*	Male	Adult	0.5	12.5	29	3.5
5731	*G. gorilla*	Male	Adult	0.48	13	28	3.8
5730	*G. gorilla*	Female	Adult	0.29	10.8	15	3
6007	*P. troglodytes*	Male	14 years	0.24	10.8	25	2
6464	*P. troglodytes*	Female	26 years	0.25	9.4	30	2.5
5428	*P. pygmaeus*	Female	Adult	0.20	8,4	21.8	1.5
Donation code [illegible]	*H. sapiens*	Male	69 years	0.36	11	31.8	3.2
Donation code [illegible]	*H. sapiens*	Female	72 years	0.38	10	29.8	3.3

SUPPLEMENTARY MATERIAL 4

Allometric scaling of echocardiographic variables and body and body size variables

In healthy modern human populations the main determinants of ARD are body size and age [10-14], including samples of athletes, where the ARD is usually within normal ranges for the general population but with larger ARD in sports with a high dynamic component [15,16]. In medicine and sports science, cardiac and circulatory variables have been normalized allometrically (by dividing the variable by some measure of body size raised to a power), but more generally ratiometrically (by dividing the variable by some measure of body size), and critical reviews have been elaborated regarding the appropriate statistical method for normalization and the most appropriate body size measure to use [15,17-20]. Fat free mass (FFM), as a measure of the metabolically active tissue, would be the most appropriate variable and, if not available, then height should be used [18]. In control [20], and athletic [15,19] samples, ARD scales ratiometrically with height whereas body surface area (BSA) only scales allometrically, with scaling exponents from 0.57 to 0.68. Other authors have used 0.5 as the scaling coefficient for BSA since ARD is a first-power measurement and BSA is a second-power measurement [12] (Supplementary Table 3). In agreement with the effect of age on ARD, the ratiometrically scaling to height is maintained only under 40 years [20]. As previously indicated [21], to assess if the scaling procedure appropriately normalizes the data, a Pearson correlation coefficient not significantly different from zero is expected between the scaled variable and the body size measure by which it was scaled. For ratiometric scaling, the Pearson correlation coefficient should also be compared to the coefficients of variation of both variables [22]. In our human sample, under 40 years of age ARD scales ratiometrically with height, with lack of correlation between ratiometrically-scaled ARD and height ($r=0.07$, $p=0.268$), and similar values for the Pearson correlation coefficient between ARD and height ($r=0.560$), and for the ratio between the coefficients of variation of both variables ($CVheight/CVARd=0.495$).

Body mass has been infrequently used for normalization of cardiac variables, and in our sample, it does not normalize ARD ratiometrically (ARD/BM and BM, $r=-0.805$, $p=0.000$; ARD and BM, $r=0.520$, $CVBM/CVARd=1.864$). However, in comparative studies in biology, BM has been the central variable used to scale physiological variables [23]. The empirically or theoretically derived scaling coefficients of ARD across diverse samples of mammal species and body masses [23-26], range from 0.25 to 0.418, as summarized in Supplementary Table 3. In our echocardiographic sample, statistical log transformations of ARD and BM and linear regression ($logARd=b \times logBM + logb$) were used to determine the scaling exponent for BM. The scaling exponent was 0.236 (95% CI: 0.199, 0.273, $R2=0.198$, $p<0.001$). As can be observed in Supplementary Table 4, in our sample only the scaling exponents 0.25 and 0.236 presented a statistically non-significant relation with body mass and thus offer an appropriate normalization of the data.

Supplementary Table 3. Scaling coefficients of ARD from several studies.

	Sample	R^2	Slope (99% CI)	Reference
**Height**	Healthy adults, rugby players		Ratiometrically	[19,20]
	Healthy adults	0.16	0.891 (0.694,1.087)	Present study
**Weight**	Mammals	0.98	0.3 (0.28,0.32)	[23]
	Mammals	0.968	0.418 (0.347,0.489)	[23]
	Humans, gorillas, chimpanzees	0.198	0.236 (0.199,0.273)	Present study
	Healthy human adults	0.163	0.244 (0.198,0.291)	Present study
**BSA**	Rugby players		0.571	[19]
	Elite athletes		0.578	[15]
	Healthy adults		0.5	[12]
	Healthy adults	0.236	0.504 (0.416,0.591)	Present study

.

Supplementary Table 4. Pearson correlation coefficient (P), and p value between body mass and ARD scaled to body mass raised to different exponents for our living hominid sample.

	**Body mass**		
**Scaled ARD**	**R**	**p**	**Reference**
$ARD / BM^{0.236}$	-0.029	0.33	Present study
$ARD / BM^{0.25}$	-0.057	0.06	[26]
$ARD / BM^{0.3}$	-0.152	< 0.001	[23]
$ARD / BM^{0.35}$	-0.242	< 0.001	[23]
$ARD / BM^{0.36}$	-0.259	< 0.001	[23]
$ARD / BM^{0.41}$	-0.352	< 0.001	[23]

SUPPLEMENTARY MATERIAL 5

Aortic root diameter and cardiac structural variables in humans and chimpanzees

A literature search was undertaken to find articles containing basic anthropometric data (sex, age, height, body mass), diameter of the aortic root and any other of the following cardiac structural variables: left ventricle end diastole diameter (LVEDD), left ventricle end systole diameter (LVESD), left ventricle end diastole volume (LVEDV), left ventricle end systole volume (LVESV), and left ventricle mass (LVMASS). If present, stroke volume (SV) was also recorded, or estimated as SV=LVEDV-LVESV. As mentioned above, one of the main determinants of ARD is age, and to avoid its effect, only samples with mean age under 30 years of age were selected. A total of 71 samples were collected from 27 studies (Supplementary File 2). The samples were classified as control (healthy subjects who do not regularly practice sports), or athlete (either professional athletes or subjects who regularly practice a specific sport). The Mitchell et al. (2005) classification scheme was also considered [27], although only the dichotomous classification control/athlete was used (20 control, 51 athlete). LVEDD and LVESV data from chimpanzees were available.

Simple linear regression analysis was used with ARD as the dependent variable, with the results summarized in Supplementary Figure 2 and Supplementary Table 5. In humans, these cardiac structural variables (LVEDD, LVESD, LVEDV, LVESV, LVMASS) statistically significantly predicted ARD. Stroke volume was also a statistically significant predictor of ARD, an expected observation in view of the results from LVEDV and LVESV. Some of these regression models presented a small sample size (LVEDV, LVESV, SV), and the analysis of the residuals from all the models indicated that a few samples exerted a strong influence on the outcomes (data not shown). If we suppress from the regression models two of the samples from [28], the parameters for both the regression models and the analyses of the residuals improved (Supplementary Figure 2, Supplementary Table 5). In chimpanzees, LVEDD and LVESD statistically significantly predicted ARD, although R^2 values were lower than in humans. This difference could be related to the fact that the human data are mean values from 71 samples with an upper age limit (30 years) to avoid the effect of age on the aortic root diameter. In chimpanzees, no upper age limit was set, and the data points shown correspond to individuals.

These results show the association of the aortic root diameter with the left ventricle in great apes, supporting its association with stroke volume, a component of the cardiac output.

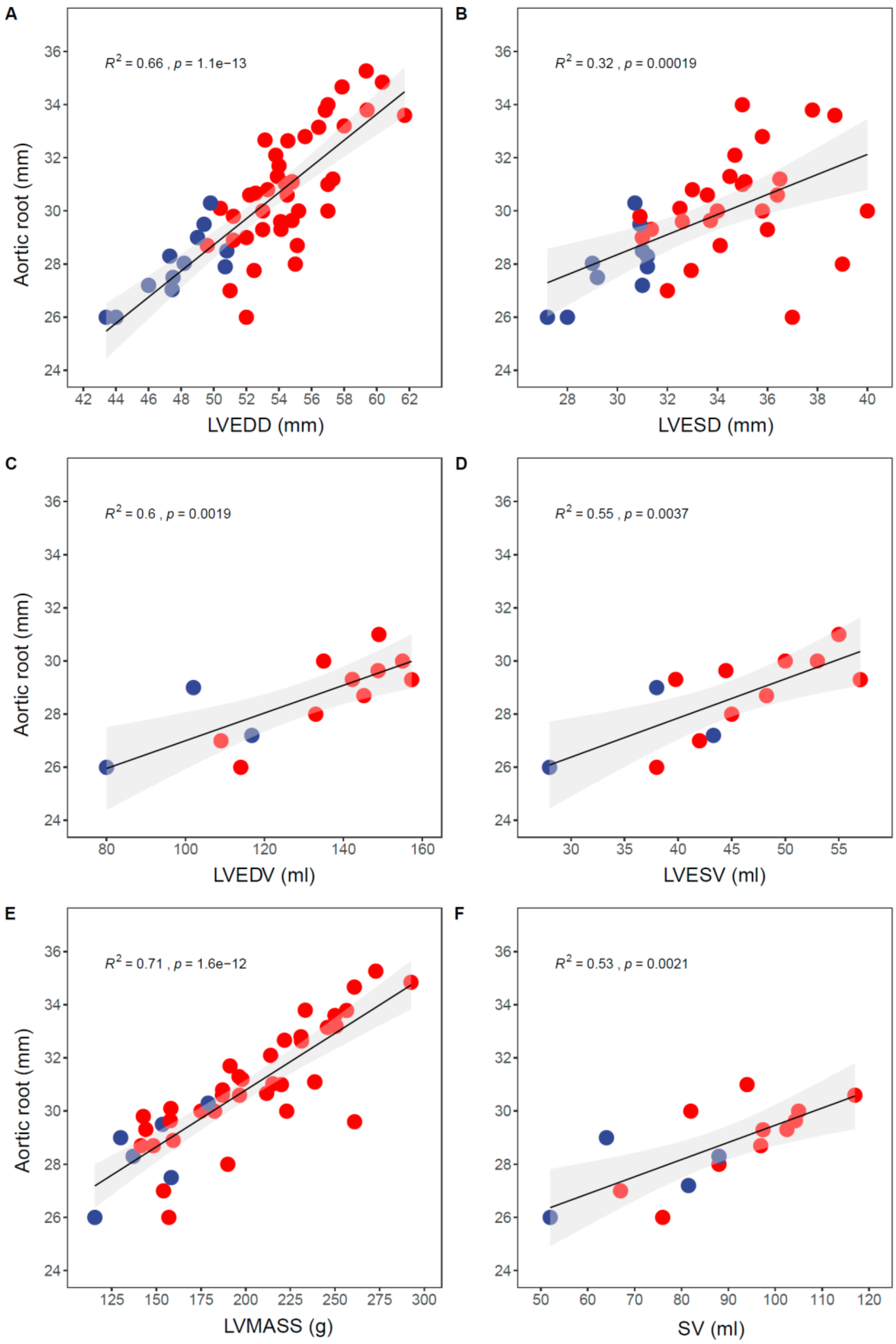

Supplementary Figure 2 (cont). Bivariate plots of ARD with five left ventricle structural variables (end diastolic diameter LVEDD, end systolic diameter LVESD, end diastolic volume EDV, end systolic volume ESV, left ventricle mass LVMASS), and with stroke volume (SV). Lines and shaded regions indicate least squares regressions and 95% confidence intervals for the control (blue) and athlete (red) total human sample, and for the pan sample (grayish blue). Adjusted R^2 and the P value are displayed for each regression model. Plots A-F correspond to the total human sample, while in plots G-L two samples from Riding et al. (2012) were excluded based on the analyses from the residuals. Plots I and J correspond to the pan values.

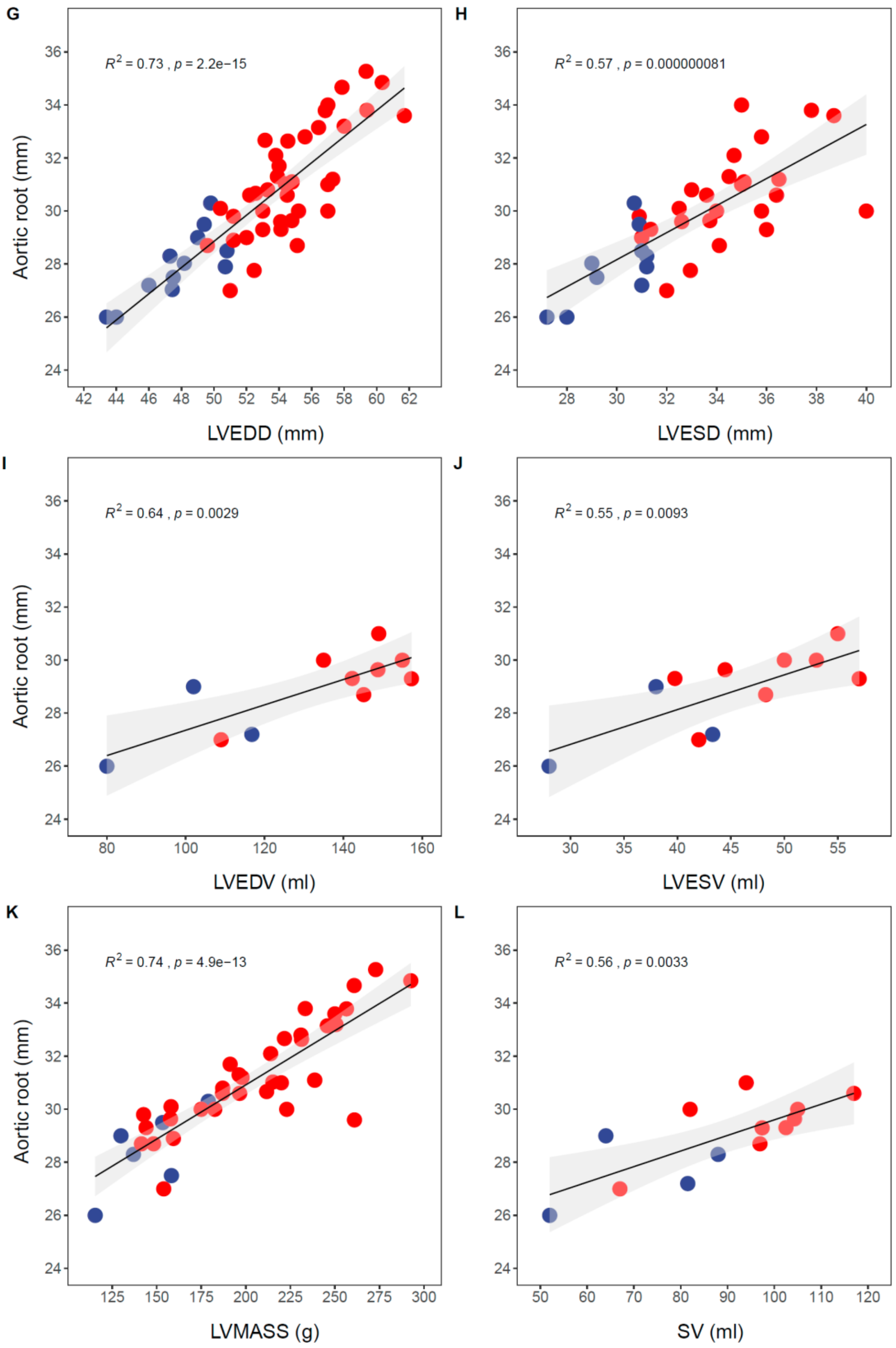

Supplementary Figure 2 (cont). Bivariate plots of ARD with five left ventricle structural variables (end diastolic diameter LVEDD, end systolic diameter LVESD, end diastolic volume EDV, end systolic volume ESV, left ventricle mass LVMASS), and with stroke volume (SV). Lines and shaded regions indicate least squares regressions and 95% confidence intervals for the control (blue) and athlete (red) total human sample, and for the pan sample (grayish blue). Adjusted R^2 and the P value are displayed for each regression model. Plots A-F correspond to the total human sample, while in plots G-L two samples from Riding et al. (2012) were excluded based on the analyses from the residuals. Plots I and J correspond to the pan values.

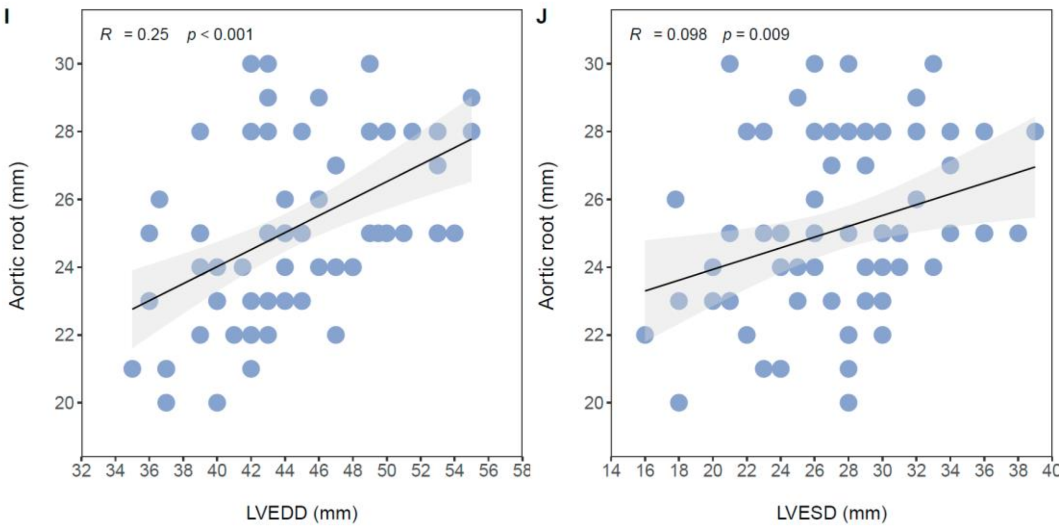

Supplementary Figure 2 (cont). Bivariate plots of ARD with five left ventricle structural variables (end diastolic diameter LVEDD, end systolic diameter LVESD, end diastolic volume EDV, end systolic volume ESV, left ventricle mass LVMASS), and with stroke volume (SV). Lines and shaded regions indicate least squares regressions and 95% confidence intervals for the control (blue) and athlete (red) total human sample, and for the pan sample (grayish blue). Adjusted R^2 and the P value are displayed for each regression model. Plots A-F correspond to the total human sample, while in plots G-L two samples from Riding et al. (2012) were excluded based on the analyses from the residuals. Plots I and J correspond to the pan values.

Supplementary Table 5. Simple linear regression models for ARD and five left ventricle structural variables (end diastolic diameter LVEDD, end systolic diameter LVESD, end diastolic volume EDV, end systolic volume ESV, left ventricle mass LVMASS), and stroke volume (SV).

**TOTAL HUMAN SAMPLE**						
	**β**	**SE**	**p**	**df**	R^2_{adj}	**SE**
**Intercept**	4.118	2.607	0.12	51	0.658	1.395
**LVEDD**	0.492	0.048	< 0.001			
**Intercept**	17.038	3.051	< 0.001	37	0.299	1.705
**LVESD**	0.377	0.09	< 0.001			
**Intercept**	21.78	1.7	< 0.001	11	0.56	1.053
**EDV**	0.052	0.0129	0.002			
**Intercept**	21.961	1.821	< 0.001	11	0.509	1.113
**ESV**	0.147	0.04	0.003			
**Intercept**	22.141	0.806	< 0.001	42	0.727	1.221
**LVMASS**	0.043	0.004	< 0.001			
**Intercept**	23.002	1.513	< 0.001	13	0.492	1.116
**SV**	0.064	0.0169	0.002			
**HUMAN SAMPLE WITHOUT 2 OUTLIERS**						
	**β**	**SE**	**p**	**df**	R^2_{adj}	**SE**
**Intercept**	4.145	2.31	0.07	49	0.72	1.232
**LVEDD**	0.494	0.043	< 0.001			
**Intercept**	12.868	2.516	< 0.001	35	0.553	1.322
**LVESD**	0.509	0.075	< 0.001			
**Intercept**	22.572	1.575	< 0.001	9	0.6051	0.945
**EDV**	0.047	0.011	0.002			
**Intercept**	22.888	1.831	< 0.001	9	0.496	1.067
**ESV**	0.131	0.039	0.009			
**Intercept**	22.551	0.731	< 0.001	40	0.7629	1.092
**LVMASS**	0.041	0.003	< 0.001			
**Intercept**	23.719	1.42	< 0.001	11	0.5199	1.018
**SV**	0.058	0.015	0.003			
**CHIMPANZEE SAMPLE**						
	**β**	**SE**	**p**	**df**	R^2_{adj}	**SE**
**Intercept**	13.978	2.386	< 0.001	67	0.240	2.283
**LVEDD**	0.251	0.053	< 0.001			
**Intercept**	20.751	1.662	< 0.001	67	0.085	2.506
**LVESD**	0.159	0.059	0.009			

SUPPLEMENTARY MATERIAL 6

Variation of cardiac output, stroke volume and heart rate in humans with age

A literature search was undertaken to find articles containing basic anthropometric data (sex, age, height, body mass), cardiac output and two or one of its components, stroke volume and heart rate. When not present, and if possible, stroke volume and cardiac output were calculated first from EDV and ESV, and then from stroke volume and heart rate respectively. Data were extracted from a total of 102 studies (Supplementary Files 3-5, references in Supplementary File 6). As explained above for the ARD, the samples were classified either as control (healthy subjects who do not regularly practice sports), or athlete (either professional athletes or subjects who regularly practice a specific sport). As mentioned before, The Mitchell et al. (2005) classification scheme was also considered [27], although only the dichotomous classification control/athlete was used.

In previous studies the normalization of cardiac output and stroke volume with height and weight has been researched [29,30], and scaling coefficients from theoretical estimations have been also used [31]. In our sample we applied and assessed these scaling coefficients and obtained and assessed sample specific allometrically and/or ratiometrically normalization of cardiac output, stroke volume and heart rate with height and weight. For the latter task, log transformations of cardiac output, stroke volume, heart rate, height and weight, and linear regression were used to determine the different scaling exponents. Results are presented in Supplementary Table 6. As explained above for the ARD, to assess the scaling procedure, Pearson correlation coefficients were obtained between the scaled variable and the body size measure by which it was scaled; and for ratiometric scaling the Pearson correlation coefficient was compared to the ratio of the coefficients of variation of both variables (Supplementary Table 7). As can be observed, there were several statistically significant correlations between the scaled-variable and either weight or height, and for the ratiometric scaling the difference between the Pearson correlation coefficient and the ratio of the coefficients of variation was also considerable for several cases. From these results, we chose to normalize cardiac output, stroke volume and heart rate by height raised to the exponents calculated for our data gathered from the literature. Also, it has been shown that fat free mass (FFM) or lean body mass, a measure of the metabolically active tissue, is strongly related to cardiac output and stroke volume [32], and to cardiac power output [33], and should be the appropriate variable to scale cardiac output and stroke volume. In absence of FFM, height would be the preferred size measure to scale cardiac variables [18].

Supplementary Table 6. Linear regression of log transformed cardiac output (CO), stroke volume (SV), heart rate (HR), height and weight, in our living hominid sample, with the respective scaling exponents.

	**Weight**					**Height**				
	R^2	R^2_{adj}	**B**	**99% CI**	**p**	R^2	R^2_{adj}	**B**	**99% CI**	**p**
**CO**	0.324	0.317	0.896	(0.558,1.234)	< 0.001	0.34	0.334	2.993	(1.893,4.094)	< 0.001
**SV**	0.354	0.348	1.059	(0.708,1.410)	< 0.001	0.507	0.503	4.159	(3.144,5.175)	< 0.001
**HR**	0.009	0.004	-0.71	(-0.200,0.058)	0.15	0.119	0.115	-0.879	(-1.289,-0.468)	< 0.001

Supplementary Table 7. Assessment of the scaling coefficients from the literature and those obtained in our sample.

**Reference**	**Scaling**	**R**	**p**	**Test CV**
[31]	CO scaled weight$^{0.75}$, quarter power law	0.063	0.52	
	SV scaled weight ratiometrically	0.000	1.0	
	HR scaled weight$^{-0.25}$, quarter power law	0.248	< 0.001	
Present study	CO scaled weight$^{0.896}$	-0.037	0.7	0.64
	SV scaled weight$^{1.059}$	0.401	< 0.001	0.58
	HR scaled weight$^{-0.71}$	0.891	0.003	0.003
	CO scaled height$^{2.993}$	-0.021	0.83	0.19
	SV scaled height$^{4.159}$	-0.005	0.95	0.17
	HR scaled height$^{-0.879}$	0.003	0.96	0.39
[30]	CO scaled weight$^{0.71}$	0.089	0.36	
	SV scaled weight$^{0.71}$	0.142	0.12	
	CO scaled height$^{1.83}$	0.167	0.09	
	SV scaled height$^{2.04}$	0.383	< 0.001	
[29]	CO scaled weight$^{0.61}$	0.161	0.10	
	CO scaled height$^{1.1}$	0.302	0.002	
	SV scaled height$^{1.45}$	0.466	< 0.001	
	HR scaled height$^{-0.33}$	-0.228	< 0.001	

Simple linear regression analysis was used to study the association between age as a predictor and the three variables, considering the type of sample (control, athlete). Results are summarized in Supplementary Table 8. Ordinary least square (OLS) regression was selected although the data were not individual cases but mean values from study samples. The criteria for the choice of the regression technique have been discussed elsewhere [34-36], between OLS and reduced major axis (RMA). Based on the considerations from these authors, we decided to use OLS.

Regarding the non-scaled variables, for the athlete samples the regression models indicate that a small percentage of the variance of cardiac output, stroke volume and heart rate is explained by age (R^2 of 0.008,

0.0052, 0.037 respectively), with statistical significance observed only for heart rate (p=0.01). For the control samples, the percentage of variation explained by age was higher for cardiac output (R^2=0.14, p=0.002) and stroke volume (R^2=0.18, p<0.001), but lower and non-statistically significant for heart rate (R^2=0.0095, p=0.29). Homogeneity of the regression slopes was tested examining the interaction between age and sample type, with lack of statistical significance for the three variables (p values 0.24, 0.09, 0.25), while difference in elevation between samples was significant in all cases (p<0.001).

Regarding the scaled variables, for both the athlete and control samples the percentages of the variance of the three variables explained by age decreased under 0.033, with statistical significance only observed for heart rate for the athlete samples (p=0.04). Again, homogeneity of the regression slopes was tested examining the interaction between age and sample type, with lack of statistical significance for the three variables (p values 0.36, 0.23, 0.23), while difference in elevation between samples was statistically non-significant for cardiac output (p=0.340), and statistically significant for stroke volume and heart rate (p<0.001). Results obtained with other scaling coefficients and body mass presented similar results (data not shown). We can conclude that cardiac output, stroke volume, and heart rate show a moderate or lack of decline with age in the athlete and control samples, especially when the variables are scaled to body size.

Differences in cardiac output, stroke volume and heart rate between control and athlete samples.

To further test the difference between control and athlete samples for the three variables, we restricted the age and height of the samples to make them comparable. The distribution of the samples across age and height shows that for cardiac output and stroke volume, the athlete samples are younger and taller, while for heart rate the height distribution is similar, but the athlete samples are also younger. As shown above, age explains a moderate but statistically significant percentage of the variation of cardiac output and stroke volume in the control samples. Also, since cardiac output and stroke volume have an allometric relation with height, the effect of the shortest samples would be stronger than the effect of the tallest samples. Thus, to avoid a possible bias of age and/or height, we restricted the samples to a maximum age of 40 years and a minimum height of 162.9 cm (the mean height of the shortest athlete sample was 163 cm). The mean difference between athlete and control samples for the non-scaled and scaled cardiac output, stroke volume and heart rate are shown in Supplementary Figure 3 [9] and Supplementary Table 9. Welch unpaired tests with Bonferroni-Holm corrections (number of tests=6) were used.

Supplementary Table 8. Simple linear regression analysis (OLS) results with age as a predictor, for the three variables cardiac output, stroke volume and heart rate (CO, SV, HR), considering the type of sample (control, athlete).

	**Non-scaled**							**Scaled**						
		**B**	**SE**	**p**	**df**	**R^2**	**SE**		**B**	**SE**	**p**	**df**	**R^2**	**SE**
**Athlete**	**Intercept**	5.787182	0.409886	< 0.001	61	0.01075	1.297	**Intercept**	0.912903	0.088129	< 0.001	54	0.003669	0.2532
	**CO**	-0.009897	0.012159	0.41				**CO / $height^{2.993}$**	0.001251	0.002806	0.65			
	**Intercept**	95.8355	5.8498	< 0.001	65	0.005247	19.06	**Intercept**	7.69163	0.65189	< 0.001	58	0.008944	1.923
	**SV**	-0.1034	0.1765	0.56				**SV / $height^{4.159}$**	0.01527	0.02111	0.47			
	**Intercept**	61.95287	1.24221	< 0.001	151	0.03685	6.628	**Intercept**	101.765	2.4829	< 0.001	130	0.03212	12.19
	**HR**	-0.08277	0.03444	0.017				**HR / $height^{0.879}$**	-0.1425	0.0686	0.039			
**Control**	**Intercept**	5.56664	0.407317	< 0.001	61	0.1389	1.189	**Intercept**	0.964434	0.077772	< 0.001	43	0.02196	0.1993
	**CO**	-0.027311	0.008706	0.002				**CO / $height^{2.993}$**	-0.001702	0.001732	0.33			
	**Intercept**	84.0799	5.397	< 0.001	70	0.1808	16.57	**Intercept**	7.33663	0.46494	< 0.001	52	0.02416	1.272
	**SV**	-0.4507	0.1147	< 0.001				**SV / $height^{4.159}$**	-0.01157	0.01019	0.26			
	**Intercept**	69.6756	1.35641	< 0.001	117	0.009522	5.466	**Intercept**	113.48839	3.2888	< 0.001	93	0.001282	11.5
	**HR**	-0.03074	0.02898	0.29				**HR / $height^{0.879}$**	-0.02413	0.06986	0.73			

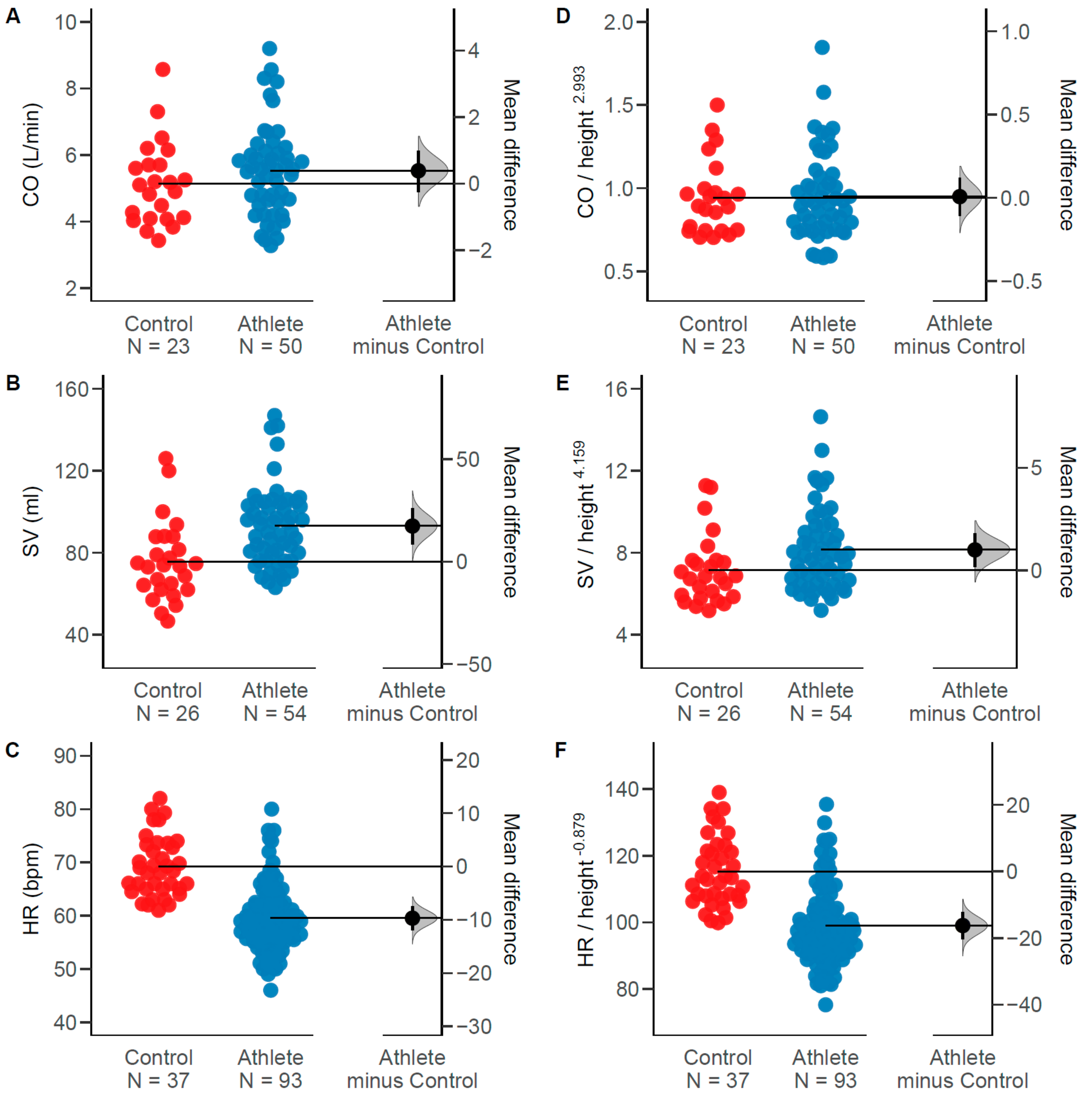

Supplementary Figure 3. Mean difference in cardiac output (CO), stroke volume (SV), and heart rate (HR) (left column, A-C), and in the same variables scaled to height (right column, D-F), between control and athlete samples. The raw data is plotted on the left axis; the mean difference is plotted on the right axis as a bootstrap sampling distribution [9]. Statistics are shown in Supplementary Table 9.

For cardiac output, no significant differences were observed between the athlete and control samples for the non-scaled and scaled variable. For stroke volume, a significant difference was observed for the non-scaled variable (P=0.00163) with a higher stroke volume in the athlete sample, while for the scaled-variables the P value was 0.0676. For heart rate, significant differences were observed between samples (P <0.001), with higher values for the control samples.

Supplementary table 9. Mean (SD) and mean difference (95% CI) for scaled and non-scaled cardiac output (CO), stroke volume (SV) and heart rate (HR), , for the restricted sample (maximum age of 40 years and a minimum height of 162.9 cm).

Variable	Control	Athlete	Mean difference (95%), p value
**CO**	5.14 (1.24)	5.53 (1.38)	0.39 (-0.26,1.04), p=0.46
**CO/height$^{2.993}$**	0.942 (0.22)	0.951 (0.26)	0.008 (-0.11,0.13), p=0.89
**SV**	75.7 (19.2)	93.1 (19.2)	17.40 (8.18,26.62), p=0.001
**SV/height4$^{.159}$**	7.14 (1.68)	8.15 (2.0)	1.00 (0.15,1.85), p=0.067
**HR**	69.2 (5.59)	59.6 (6.37)	-9.67 (56.55,69.22), p<0.001
**HR/height$^{-0.879}$**	115 (10.4)	99.1 (11.8)	-16.22 (-20.34,-12.04), p<0.001

SUPPLEMENTARY MATERIAL 7

Variation across lifespan of cardiac output, total energy expenditure, organs' weight, and anthropometry

A literature search was undertaken to find articles containing data on cardiac output, organ weight, height and weight, and TEE, across the life span or along the growth period. For cardiac output, results from research conducted on a local, healthy sample from Hong Kong, aged 0-60 years were selected [37-40]. Anthropometric data from this study was available below the age of 17 years, and for this subsample, cardiac output was adjusted using the exponents calculated previously for height and body surface area[30]. When considering the whole lifespan, since anthropometric data was not available, cardiac output adjusted to body surface area (bsa) was used (cardiac output/bsa)[37]. For organ weight, and taking into account the previously selected Hong Kong sample for cardiac output, results from a cadaver study on a nationwide Japanese sample were selected [41]. For height and weight, for illustrative purposes, and due to its completeness, data from the CDC Growth Charts were also selected [42]. The objective of presenting anthropometric data was to compare its pattern of change with age with the patterns from the other variables (cardiac output, TEE, organs) (Figure 3, Supplementary Figure 4). For TEE, results obtained through the doubly labeled water method were selected, obtained in subjects aged 8-95 years [43]. (Supplementary Files 7-12).

Basic data were extracted from the article's tables and figures. Data from figures were extracted with webplotdigitizer (https://automeris.io/WebPlotDigitizer/). In order to compare data from different variables, we followed a procedure described previously [44]. Continuous functions were fit separately to all the variables (absolute and adjusted cardiac output and TEE, organs weight, height, and weight), and different models were evaluated using the Akaike Information Criteria (AIC), for the age range 0-19 or 0-27 years. A Gompertz model was selected for brain weight growth, while for the other variables, a cubic spline function with four knots was selected, placed at age quantiles. Once the models were selected, predicted values were calculated at 0.2 years intervals for all the variables. Z-scores and derived variables (velocity) were then calculated from those predicted variables, and the comparative graphs were built.

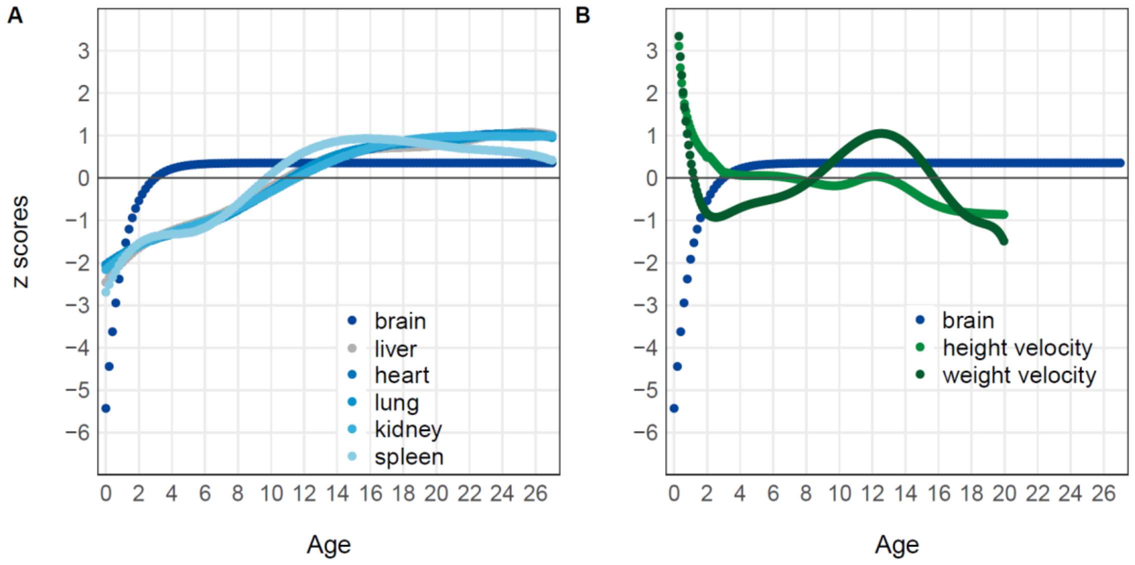

Supplementary Figure 4. Change from birth to adulthood of brain weight and organs weight (A), and change from birth to adulthood of brain weight, together with the velocity curve for height and weight (B). Original data were first obtained from the literature, and then a Gompertz (brain), or cubic spline functions (all the other organs), were fitted to the values. Predicted values from the functions were obtained (first derivative was also calculated for height and weight) and converted to z-scores for comparison between variables.

SUPPLEMENTARY MATERIAL 8

Aortic impression

The skeletal sample is shown by genera and sex in Supplementary Table 10. Great ape specimens came from the Africa Museum (Tervuren, Belgium), Museo Anatómico (Faculty of Medical Sciences, Universidad de Valladolid, Spain), Estación Biológica de Doñana (EBD-CSIC, Sevilla, Spain), and Museu de Ciències Naturals (Barcelona, Spain). Modern human skeletons belong to the Luís Lopes Anthropological Collection, MUHNAC (Lisbon, Portugal). Two additional fossil specimens were included, a Neanderthal vertebra from El Sidrón (Asturias, Spain), curated by Antonio Rosas at the Museo Nacional de Ciencias Naturales (MNCN-CSIC, Madrid, Spain), and images from the vertebrae of KNM WT 15000, from 3D surface scans obtained from the original fossil by Markus Bastir (Nairobi, National Museums of Kenya).

Supplementary Table 10. Samples studied by genus and sex.

	Female	Male	Total
**Humans**	24	24	48
Pan	22	21	43
Gorilla	12	12	24
Pongo	5	1	6
**Total**	63	58	121

For each specimen, first, the available vertebrae were articulated from C1 (first cervical) to L4 (great apes), or L5 (modern humans). The vertebrae had to articulate smoothly, with a clear fitting of the area and perimeter of the body, as well as the superior and inferior articular surfaces, between adjacent vertebrae. A correct change of shape of the spinous process along the spine was also checked, as indicative of a correct vertebral sequence. Vertebrae with postmortem erosion or degenerative disease were excluded, and in the case of the human sample, this exclusion was also based on the selection of a young adult sample (under 30 years). After this process, the vertebrae from the specimen were considered ready to study. Due to the different vertebral formulae, we chose to name vertebral levels following a continuous numeration from superior (V1= C1 or first cervical vertebra) to inferior [45].

Photographs were taken with a Canon EOS 70D with ef-s 18-135mm f/3.5-5.6 lenses. Each vertebra from T1 to L4 was positioned with the inferior vertebral body surface parallel to the lens of the camera, placed at a fixed distance, with the identification of the specimen and the corresponding vertebral number, and with a scale in cm. The background was uniform black or white. The position of each vertebra was monitored directly and through the grids of the articulated LCD touchscreen of the camera to fit the following three

spatial criteria: the spinous process was situated superior and the anterior border of the epiphyseal ring inferior, a straight line including the left and right most posterior points of the epiphyseal ring was parallel to the long axis of the image, a straight line including both the most posterior and most anterior points within the vertebral canal was parallel to the short axis of the image.

Each photo was transferred to Adobe Photoshop (CS6) where, if needed, its position was rotated to further fit the previous criteria. Several vertebrae were asymmetrical and did not fit all these criteria simultaneously. In these cases, the alignment of the vertebral image was carried out trying to maximize the correct alignment of the vertebral endplate. The rectangle tool was used to display a rectangle whose superior side contacted the posterior border of the epiphyseal ring, and whose inferior side contacted the most anterior point of the epiphyseal ring. The lateral sides of the rectangle contacted the left and right points of intersection between the epiphyseal ring and the costal demi-facet (upper thoracic vertebrae), or the left and right most lateral points of the epiphyseal ring (middle thoracic to lumbar vertebrae). With the rectangle in place, the surface of the body was colored uniformly and the midpoints of the four sides of the rectangle were automatically displayed. The rectangle was divided into quadrants by two perpendicular lines passing through those four midpoints.

This image was saved and transferred to ImageJ [46], and after setting the scale, the surface areas of the anterior left and right quadrants were determined. These were the values used for the calculation of the asymmetry. The potential error associated to this procedure was tested by repeating the protocol again for vertebrae from 10 cases (n=158), with an intraclass correlation coefficient of 0.998, and relative technical error of measurement of 2.07 [47]. Asymmetry was calculated for each vertebra following a formula for relative asymmetry that standardizes to within-individual percentages, with positive values indicating a larger right side [48] (Supplementary Files 13,14). Parametric (one sample t-test, independent samples t-test) and non-parametric (Wilcoxon signed rank, independent samples Mann-Whitney U) tests were applied for detecting significant asymmetry in each vertebra, and genus-based differences in asymmetry. The results from both types of tests were identical in the statistical significance per vertebral level, and results from the parametric tests are shown in Supplementary Table 11.

Supplementary Table 11. P values from the parametric tests for vertebral asymmetry per vertebral level (one sample t test), and differences in vertebral asymmetry between modern humans and great apes (two independent samples t test).

Vertebral level	One sample t test p values		Two independent samples t test p values
	Modern humans	Great apes	Humans vs Great apes
**V8**	0.44	0.44	0.92
**V9**	0.42	0.14	0.91
**V10**	0.001	0.008	0.04
**V11**	0.61	0.74	0.55
**V12**	0.029	0.45	0.07
**V13**	0.001	0.21	0.01
**V14**	<0.001	0.77	<0.001
**V15**	<0.001	0.01	<0.001
**V16**	<0.001	0.02	<0.001
**V17**	<0.001	0.003	<0.001
**V18**	<0.001	<0.001	<0.001
**V19**	<0.001	<0.001	0.88
**V20**	0.002	<0.001	0.47
**V21**	<0.001	0.06	0.08
**V22**	0.57	0.48	0.38
**V23**	0.03	0.71	0.11

It is important to address the limitations of the assumption of a direct relationship between bilateral asymmetry of the anterior half of the vertebral body and the presence of an aortic impression, defined as "a variable flattening that may be found on the left side of the bodies of mid-thoracic vertebrae" [49]. This caution is warranted since clear cases of asymmetry of individual vertebrae can be observed in Figure 4A. The vertebral column is a metameric structure that expresses information related to ontogeny and ageing, body mass, posture, and locomotion, breathing kinematics and other physiological functions related to thoracic and abdominal organs. The asymmetry measured in the present study captures more asymmetry than that strictly related to the aortic impression. As an additional check, each image was visually assessed to detect any asymmetry caused by a unilateral flattening on the left side of the body: no great ape vertebra presented this asymmetry. Finally, since the trajectories of asymmetry along the vertebral column of individuals is not smooth, where a complete vertebral column is lacking, absence of asymmetry and thus of an aortic impression in an isolated vertebra should be considered with caution.

SUPPLEMENTARY MATERIAL 9

Heart rate

In a large national sample from the USA, the NHANES I, [50] observed that for adults, supine resting heart rate (RHR, measured by electrocardiogram) was not associated to height or weight in most subgroups (sex, age, ancestry). Age and ancestry were also not associated to RHR, but a consistently higher RHR was observed in women (average of 3 bpm) that was not explained by the measured covariates. Similar findings were obtained for resting radial pulse rate (RPR, measured manually) in the same sample [51]. In the latter study, slower RPR were observed in more active persons, with difference of 6-8 bpm for males and 1-4 bpm for females. A more recent study of the NHANES 1999-2008 data [52], found a small by significant effect of ancestry on RPR, and a moderate change of RPR with age in adults. A mean difference of 3 bpm more in females than males was also observed in these studies (and in our database, 3.6 bpm, see Supplementary Figure 5A), difference that augments during pregnancy by 7.6 bpm as shown by Loerup et al. (2019) [53] in a meta-analysis of 39 previous studies. In the study of Peruvian people living across a gradient of 5000 m of altitude, negligible variations of RHR (measured by a calibrated pulse oximeter), were observed according to the altitude of residence in the four age groups studied (1-5, 6-17, 18-50, 51-80 years) [54]. In our database we collected 153 athlete or physically active samples and 121 healthy control samples from 96 studies, from 17.5 to 75 years old. In Supplementary Figure 6, the variation with age of heart rate and heart rate scaled to weight raised to the power -0.25 for these athlete/control samples is displayed, with negligible or statistically not significant associations. With relation to physical activity, the mean difference in heart rate between athlete and control samples was 9.02 bpm lower in the athlete sample (Supplementary Figure 5B), especially for those involved in endurance training.

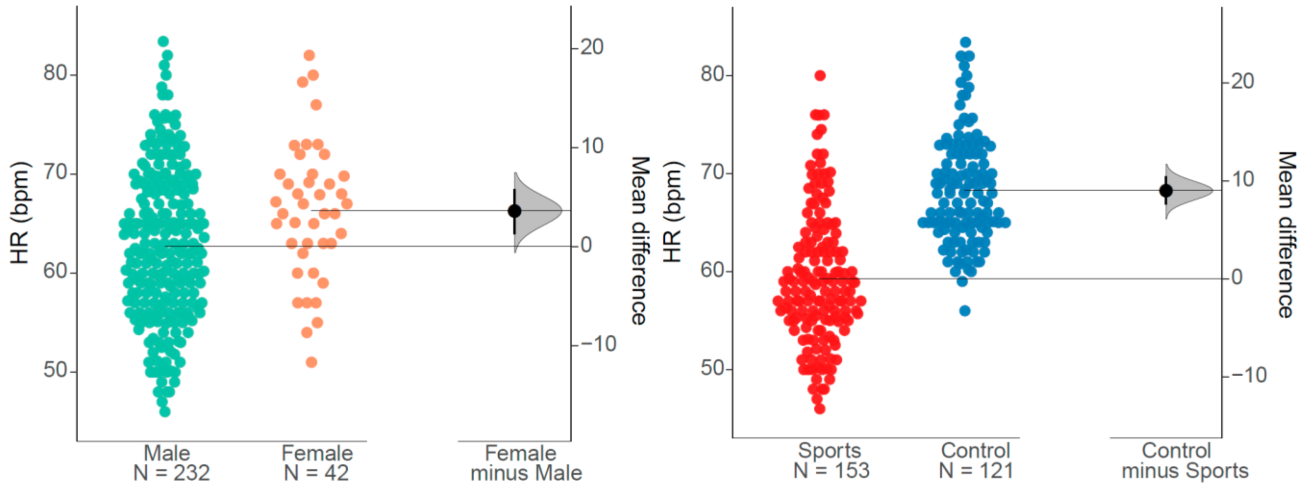

Supplementary Figure 5. Mean difference in heart rate between males and females (A), and athlete and control samples (B). In both cases, groups are plotted on the left axes, while the mean difference is plotted on a floating axis on the right as a bootstrap sampling distribution. The raw data is plotted on the left axis; the mean difference is plotted on the right axis as a bootstrap sampling distribution [9].

.

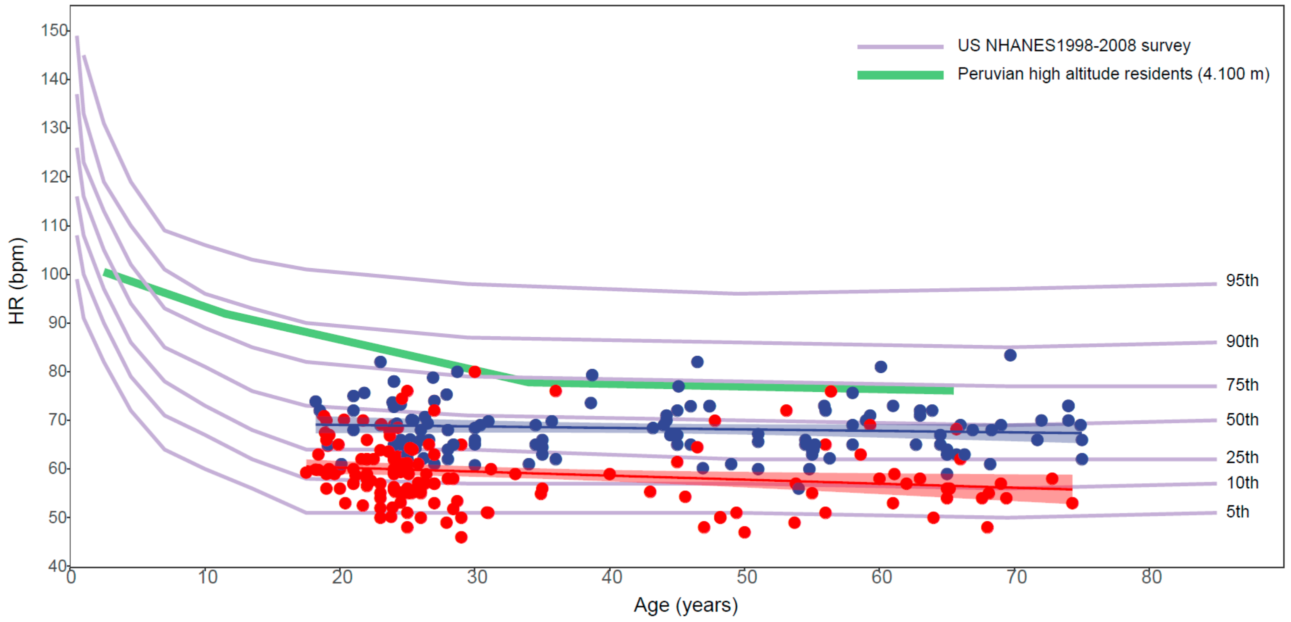

Supplementary Figure 6. Percentile values for the US sample (males and females) from the 1999-2008 National Health and Nutrition Examination Surveys studied by Ostchega et al. (2011), obtained from their Table 1. The mean values from Peruvians residents at the high-altitude location of Junín (4.100 m) [54], obtained from their Table 1, is also included. Red and blue circles represent respectively athlete and healthy control samples. Lines and shaded regions indicate least squares regressions and 95% confidence intervals for both samples.

REFERENCES

1 Bogin, B. *Patterns of human growth*. Vol. 88 (Cambridge University Press, 2020).

2 Bolter, D. R. & Zihlman, A. L. Skeletal development in Pan paniscus with comparisons to Pan troglodytes. *American journal of physical anthropology* **147**, 629-636 (2012).

3 Zihlman, A. L., Bolter, D. R. & Boesch, C. Skeletal and dental growth and development in chimpanzees of the Taï National Park, Côte D'Ivoire. *Journal of Zoology* **273**, 63-73 (2007).

4 Galbany, J. *et al.* Body growth and life history in wild mountain gorillas (Gorilla beringei beringei) from Volcanoes National Park, Rwanda. *American Journal of Physical Anthropology* **163**, 570-590 (2017).

5 Hamada, Y. & Udono, T. Longitudinal analysis of length growth in the chimpanzee (Pan troglodytes). *American Journal of Physical Anthropology: The Official Publication of the American Association of Physical Anthropologists* **118**, 268-284 (2002).

6 Pusey, A. E., Oehlert, G. W., Williams, J. & Goodall, J. Influence of ecological and social factors on body mass of wild chimpanzees. *International Journal of Primatology* **26**, 3-31 (2005).

7 Machanda, Z. *et al.* MUSCULOSKELETAL GROWTH IN WILD CHIMPANZEES WITH IMPLICATIONS FOR SOCIAL BEHAVIOR. *American Journal of Primatology* **80** (2018).

8 Armstrong, R. A. When to use the Bonferroni correction. *Ophthalmic and Physiological Optics* **34**, 502-508, doi:10.1111/opo.12131 (2014).

9 Ho, J., Tumkaya, T., Aryal, S., Choi, H. & Claridge-Chang, A. Moving beyond P values: data analysis with estimation graphics. *Nature Methods* **16**, 565-566, doi:10.1038/s41592-019-0470-3 (2019).

10 Biaggi, P. *et al.* Gender, Age, and Body Surface Area are the Major Determinants of Ascending Aorta Dimensions in Subjects With Apparently Normal Echocardiograms. *Journal of the American Society of Echocardiography* **22**, 720-725, doi:10.1016/j.echo.2009.03.012 (2009).

11 Campens, L. *et al.* Reference Values for Echocardiographic Assessment of the Diameter of the Aortic Root and Ascending Aorta Spanning All Age Categories. *American Journal of Cardiology* **114**, 914-920, doi:10.1016/j.amjcard.2014.06.024 (2014).

12 Devereux, R. B. *et al.* Normal Limits in Relation to Age, Body Size and Gender of Two-Dimensional Echocardiographic Aortic Root Dimensions in Persons >= 15 Years of Age. *American Journal of Cardiology* **110**, 1189-1194, doi:10.1016/j.amjcard.2012.05.063 (2012).

13 Vasan, R. S., Larson, M. G. & Levy, D. DETERMINANTS OF ECHOCARDIOGRAPHIC AORTIC ROOT SIZE - THE FRAMINGHAM HEART-STUDY. *Circulation* **91**, 734-740, doi:10.1161/01.Cir.91.3.734 (1995).

14 Vriz, O. *et al.* Normal Values of Aortic Root Dimensions in Healthy Adults. *American Journal of Cardiology* **114**, 921-927, doi:10.1016/j.amjcard.2014.06.028 (2014).

15 Abuli, M. *et al.* Aortic root remodelling in competitive athletes. *European Journal of Preventive Cardiology* **27**, 1518-1526, doi:10.1177/2047487319894882 (2020).

16 Boraita, A. *et al.* Reference Values of Aortic Root in Male and Female White Elite Athletes According to Sport. *Circulation-Cardiovascular Imaging* **9**, doi:10.1161/circimaging.116.005292 (2016).

17 Batterham, A. M., George, K. P., Whyte, G., Sharma, S. & McKenna, W. Scaling cardiac structural data by body dimensions: A review of theory, practice, and problems. *International Journal of Sports Medicine* **20**, 495-502 (1999).

18 Dewey, F. E., Rosenthal, D., Murphy, D. J., Jr., Froelicher, V. F. & Ashley, E. A. Does size matter? Clinical applications of scaling cardiac size and function for body size. *Circulation* **117**, 2279-2287, doi:10.1161/circulationaha.107.736785 (2008).

19 Oates, S. A. *et al.* Scaling to produce size-independent indices of echocardiographic derived aortic root dimensions in elite Rugby Football League players. *Ultrasound* **27**, 94-100, doi:10.1177/1742271x18818607 (2019).

20 Oxborough, D. *et al.* Impact of methodology and the use of allometric scaling on the echocardiographic assessment of the aortic root and arch: a study by the Research and Audit Sub-Committee of the British Society of Echocardiography. *Echo research and practice* **1**, 1-9, doi:10.1530/erp-14-0004 (2014).

21 Turley, K. R. *et al.* Scaling submaximal exercise cardiac output and stroke volume: The HERITAGE family study. *International Journal of Sports Medicine* **27**, 993-999, doi:10.1055/s-2006-923835 (2006).
22 Tanner, J. M. THE CONSTRUCTION OF NORMAL STANDARDS FOR CARDIAC OUTPUT IN MAN. *American Journal of the Medical Sciences* **216**, 598-599 (1948).
23 Prothero, J. W. *The design of mammals*. (Cambridge University Press, 2015).
24 Bengtsson, H. U. & Eden, P. A simple model for the arterial system. *Journal of Theoretical Biology* **221**, 437-443, doi:10.1006/jtbi.2003.3198 (2003).
25 Holt, J. P., Rhode, E. A., Holt, W. W. & Kines, H. GEOMETRIC SIMILARITY OF AORTA, VENAE CAVAE, AND CERTAIN OF THEIR BRANCHES IN MAMMALS. *American Journal of Physiology* **241**, R100-R104, doi:10.1152/ajpregu.1981.241.1.R100 (1981).
26 West, G. B., Brown, J. H. & Enquist, B. J. A general model for the origin of allometric scaling laws in biology. *Science* **276**, 122-126, doi:10.1126/science.276.5309.122 (1997).
27 Mitchell, J. H., Haskell, W., Snell, P. & Van Camp, S. P. Task Force 8: classification of sports. *Journal of the American College of Cardiology* **45**, 1364-1367 (2005).
28 Riding, N. R. *et al.* Do big athletes have big hearts? Impact of extreme anthropometry upon cardiac hypertrophy in professional male athletes. *British journal of sports medicine* **46**, i90-i97 (2012).
29 Chirinos, J. A. *et al.* Time-Varying Myocardial Stress and Systolic Pressure-Stress Relationship Role in Myocardial-Arterial Coupling in Hypertension. *Circulation* **119**, 2798-U2100, doi:10.1161/circulationaha.108.829366 (2009).
30 deSimone, G. *et al.* Stroke volume and cardiac output in normotensive children and adults - Assessment of relations with body size and impact of overweight. *Circulation* **95**, 1837-1843 (1997).
31 Shave, R. E. *et al.* Selection of endurance capabilities and the trade-off between pressure and volume in the evolution of the human heart. *Proceedings of the National Academy of Sciences of the United States of America* **116**, 19905-19910, doi:10.1073/pnas.1906902116 (2019).
32 Collis, T. *et al.* Relations of stroke volume and cardiac output to body composition - The strong heart study. *Circulation* **103**, 820-825, doi:10.1161/01.Cir.103.6.820 (2001).
33 Chantler, P. D. *et al.* The influence of body size on measurements of overall cardiac function. *American Journal of Physiology-Heart and Circulatory Physiology* **289**, H2059-H2065, doi:10.1152/ajpheart.00022.2005 (2005).
34 Kilmer, J. T. & Rodriguez, R. L. Ordinary least squares regression is indicated for studies of allometry. *Journal of Evolutionary Biology* **30**, 4-12, doi:10.1111/jeb.12986 (2017).
35 Smith, R. J. Use and Misuse of the Reduced Major Axis for Line-Fitting. *American Journal of Physical Anthropology* **140**, 476-486, doi:10.1002/ajpa.21090 (2009).
36 White, C. R. & Kearney, M. R. Metabolic Scaling in Animals: Methods, Empirical Results, and Theoretical Explanations. *Comprehensive Physiology* **4**, 231-256, doi:10.1002/cphy.c110049 (2014).
37 Cattermole, G. N. *et al.* The normal ranges of cardiovascular parameters measured using the ultrasonic cardiac output monitor. *Physiological Reports* **5**, doi:10.14814/phy2.13195 (2017).
38 Cattermole, G. N. *et al.* The normal ranges of cardiovascular parameters in children measured using the Ultrasonic Cardiac Output Monitor. *Critical Care Medicine* **38**, 1875-1881, doi:10.1097/CCM.0b013e3181e8adee (2010).
39 Ho, G. Y. L. *et al.* Noninvasive Transcutaneous Doppler Ultrasound-Derived Hemodynamic Reference Ranges in Chinese Adolescents. *Pediatric Critical Care Medicine* **14**, E225-E232, doi:10.1097/PCC.0b013e3182772f78 (2013).
40 Rainer, T. H., Cattermole, G. N., Graham, C. A. & Chan, S. S. W. Anthropometric and physiological measurements in healthy children. *Hong Kong medical journal = Xianggang yi xue za zhi* **19 Suppl 9**, 26-29 (2013).
41 Ogiu, N., Nakamura, Y., Ijiri, I., Hiraiwa, K. & Ogiu, T. A statistical analysis of the internal organ weights of normal Japanese people. *Health Physics* **72**, 368-383, doi:10.1097/00004032-199703000-00004 (1997).

42 Kuczmarski, R. J. *et al.* 2000 CDC Growth Charts for the United States: methods and development. *Vital and health statistics. Series 11, Data from the National Health Survey*, 1-190 (2002).
43 Pontzer, H. *et al.* Daily energy expenditure through the human life course. *Science* **373**, 808-+, doi:10.1126/science.abe5017 (2021).
44 Kuzawa, C. W. *et al.* Metabolic costs and evolutionary implications of human brain development. *Proceedings of the National Academy of Sciences of the United States of America* **111**, 13010-13015, doi:10.1073/pnas.1323099111 (2014).
45 Williams, S. A., Gómez-Olivencia, A. & Pilbeam, D. R. in *Spinal Evolution* 97-124 (Springer, 2019).
46 Schneider, C. A., Rasband, W. S. & Eliceiri, K. W. NIH Image to ImageJ: 25 years of image analysis. *Nature Methods* **9**, 671-675, doi:10.1038/nmeth.2089 (2012).
47 Ayele, B. *et al.* Reliability of Measurements Performed by Community-Drawn Anthropometrists from Rural Ethiopia. *Plos One* **7**, doi:10.1371/journal.pone.0030345 (2012).
48 Plochocki, J. H. Bilateral variation in limb articular surface dimensions. *American Journal of Human Biology* **16**, 328-333, doi:10.1002/ajhb.20023 (2004).
49 White, T. D., Black, M. T. & Folkens, P. A. *Human osteology*. (Academic press, 2011).
50 Gillum, R. F. THE EPIDEMIOLOGY OF RESTING HEART-RATE IN A NATIONAL SAMPLE OF MEN AND WOMEN - ASSOCIATIONS WITH HYPERTENSION, CORONARY HEART-DISEASE, BLOOD-PRESSURE, AND OTHER CARDIOVASCULAR RISK-FACTORS. *American Heart Journal* **116**, 163-174, doi:10.1016/0002-8703(88)90262-1 (1988).
51 Gillum, R. F. EPIDEMIOLOGY OF RESTING PULSE-RATE OF PERSONS AGES 25-74 - DATA FROM NHANES 1971-74. *Public Health Reports* **107**, 193-201 (1992).
52 Ostchega, Y., Porter, K. S., Hughes, J., Dillon, C. F. & Nwankwo, T. Resting pulse rate reference data for children, adolescents, and adults: United States, 1999-2008. *National health statistics reports*, 1-16 (2011).
53 Loerup, L. *et al.* Trends of blood pressure and heart rate in normal pregnancies: a systematic review and meta-analysis. *Bmc Medicine* **17**, doi:10.1186/s12916-019-1399-1 (2019).
54 Mejia, C. R. *et al.* Values of heart rate at rest in children and adults living at different altitudes in the Andes. *Plos One* **14**, doi:10.1371/journal.pone.0213014 (2019).
