# Supplementary material for: The aorta in humans and African great apes, and cardiac output and metabolic levels in human evolution
Source: Sci Rep. 2023 Apr 26;13:6841. doi: 10.1038/s41598-023-33675-1 (PMC10133235; doi:10.1038/s41598-023-33675-1)
Supplement: Supplementary file 2 — Supplementary Information 2. [file 41598_2023_33675_MOESM2_ESM.pdf]

## Equivalence supplementary file name and dataframes used:

| | |
|---|---|
| Supplementary_file_1 | ARD |
| Supplementary_file_2 | ARDCARDIAC |
| Supplementary_file_3 | CO |
| Supplementary_file_4 | HR |
| Supplementary_file_5 | SV |
| Supplementary_file_7 | figure3a |
| Supplementary_file_8 | figure3b |
| Supplementary_file_9 | figure3c |
| Supplementary_file_10 | figure3d |
| Supplementary_file_11 | organs |
| Supplementary_file_12 | growthmfhwbzs |
| Supplementary_file_13 | humans |
| Supplementary_file_14 | greatapes |

```
# figure 1, rain cloud graphs for aorta, body mass,
and aorta scaled body mass 0.236

a_scl <-ggplot(ARD,aes(x=species,y=a0236, fill=species,
colour=species)) +
ylim(6,16)+geom_flat_violin(position=position_nudge (x=.25, y=0),
adjust=1, trim=FALSE) +
geom_point(position=position_jitter(width=.15), size=1.5)+
geom_boxplot(aes(x=as.numeric(species)+0.25,
y=a0236),outlier.shape=NA, alpha=0.3, width=.1, colour="BLACK")+
labs(title=NULL, y="Aortic root (mm)/mass-0.236", x = NULL)+
coord_flip()+
theme_cowplot()+
theme(axis.title.y=element_blank(), axis.title.x=element_blank(),
axis.text.x=element_blank(), plot.margin = margin(0.5, 0.5, 0.5,
0.5, "cm"), axis.ticks.length.x= unit(-0.2, "cm"))+
guides(fill = FALSE, colour = FALSE)+
scale_colour_brewer(palette = "Set2", direction=-1)+
scale_fill_brewer(palette = "Set2", direction=-1)
as<- a_scl + annotate("text",  x = 0.51, y = c(6,8,10,12,14,16),
label = c("6", "8", "10", "12","14", "16"), size=4)+annotate("text",
x = 0.67, y = 13.5, label = "Aortic root (mm)/mass-0.236", size=4)

a_raw<-ggplot(ARD,aes(x=species,y=aorta, fill=species,
colour=species)) + ylim(18,50)+
geom_flat_violin(position=position_nudge (x=.25, y=0), adjust=1,
trim=FALSE)+  geom_point(position=position_jitter(width=.15),
size=1.5) +
geom_boxplot(aes(x=as.numeric(species)+0.25,
y=aorta),outlier.shape=NA, alpha=0.3, width=.1, colour="BLACK")+
labs(title=NULL, y="Aortic root (mm)")+
theme_cowplot()+
```

```
theme(axis.title.y=element_blank(), axis.text.y=element_blank(),
axis.ticks.y=element_blank(), axis.title.x=element_blank(),
axis.text.x=element_blank(), plot.margin = margin(0.5, 0.5, 0.5,
0.5, "cm"), axis.ticks.length.x= unit(-0.2, "cm"))+
coord_flip()+
scale_y_continuous(expand = c(0, 0))+
scale_x_discrete(expand = c(0.2,0.2))+
guides(fill = FALSE, colour = FALSE)+
scale_colour_brewer(palette = "Set2", direction=-1)+
scale_fill_brewer(palette = "Set2", direction=-1)
ar<-a_raw+annotate("text",  x = 0.65, y = c(20,30,40,50), label =
c("20", "30", "40", "50"), size=4)+annotate("text", x = 1, y = 44,
label = "Aortic root (mm)", size=4)

mass_raw <-ggplot(ARD,aes(x=species,y=bodymass, fill=species,
colour=species))+
geom_flat_violin(position=position_nudge (x=.25, y=0), adjust=1,
trim=FALSE)+  geom_point(position=position_jitter(width=.15),
size=1.5)+
geom_boxplot(aes(x=as.numeric(species)+0.25, y=bodymass),
outlier.shape=NA, alpha=0.3, width=.1, colour="BLACK")+
labs(title=NULL, y="Body mass (kg)")+
theme_cowplot()+
theme(axis.title.y=element_blank(), axis.text.y=element_blank(),
axis.ticks.y=element_blank(), axis.title.x=element_blank(),
axis.text.x=element_blank(), plot.margin = margin(0.5, 0.5, 0.5,
0.5, "cm"), axis.ticks.length.x= unit(-0.2, "cm"))+
coord_flip()+
scale_y_continuous(expand = c(0, 0))+
scale_x_discrete(expand = c(0.2,0.2))+
guides(fill = FALSE, colour = FALSE)+
scale_colour_brewer(palette = "Set2", direction=-1)+
scale_fill_brewer(palette = "Set2", direction=-1)
```

```
mr<-mass_raw+annotate("text",  x = 0.65, y = c(100,200,300), label =
c("100", "200", "300"),size=4)+annotate("text", x = 1, y = 250,
label = "Body mass (kg)", size=4)

lay <- rbind(c(1,2), c(1,3))
figure1<- grid.arrange(as,ar,mr, layout_matrix=lay, widths=c(2,1))
ggsave(file="figure1.pdf", figure1, width=9, height=6)
```

# # figure 2, change with age of CO, SV, and HR, non-scaled and scaled

```
co<-ggplot(CO, aes(x=age, y=CO))+
theme_bw()+
theme(panel.grid.major=element_blank(),
panel.grid.minor=element_blank())+
theme(legend.position="none")+
geom_point(aes(colour=sample), size=4)+
stat_smooth(aes(color = sample, fill = sample), method = "lm")+
scale_color_aaas(palette = c("default"), alpha = 1)+
scale_fill_aaas(palette = c("default"), alpha = 1)+
scale_x_continuous(expand=c(0,0), limits=c(15,85),
breaks=c(20,30,40,50,60,70,80))+ scale_y_continuous(ylab("CO
(L/min)"), limits=c(2,9),breaks=c(2,3,4,5,6,7,8,9))+
stat_cor(aes(color = sample, label=paste
(..rr.label..,..p.label..,sep = " ~`,`~")), label.x=66, size=3.5)+
theme(axis.title.x=element_blank(), axis.text.x = element_text(size
= 12), axis.title.y = element_text(size = 14), axis.text.y =
element_text(size = 12))

cosc<-ggplot(CO, aes(x=age, y= CO_height2993))+
theme_bw()+
```

```
theme(panel.grid.major=element_blank(),
panel.grid.minor=element_blank())+
theme(legend.position="none")+
geom_point(aes(colour=sample), size=4)+
stat_smooth(aes(color = sample, fill = sample), method = "lm")+
scale_color_aaas(palette = c("default"), alpha = 1)+
scale_fill_aaas(palette = c("default"), alpha = 1)+
scale_x_continuous(expand=c(0,0), limits=c(15,85),
breaks=c(20,30,40,50,60,70,80))+ scale_y_continuous(ylab("CO
(L/min)/height2.993 "), limits=c(0.5,2.2),breaks=c(0.6, 1, 1.4, 1.8,
2.2))+
stat_cor(aes(color = sample, label=paste
(..rr.label..,..p.label..,sep = " ~`,`~")), label.x=66, size=3.5)+
theme(axis.title.x=element_blank(), axis.text.x = element_text(size
= 12), axis.title.y = element_text(size = 14), axis.text.y =
element_text(size = 12))

sv<-ggplot(SV, aes(x=age, y=SV))+
theme_bw()+
theme(panel.grid.major=element_blank(),
panel.grid.minor=element_blank())+
theme(legend.position="none")+
geom_point(aes(colour=sample), size=4)+
stat_smooth(aes(color = sample, fill = sample), method = "lm")+
scale_color_aaas(palette = c("default"), alpha = 1)+
scale_fill_aaas(palette = c("default"), alpha = 1)+
scale_x_continuous(expand=c(0,0), limits=c(15,85),
breaks=c(20,30,40,50,60,70,80))+
scale_y_continuous(ylab("SV (ml)"),
limits=c(30,160),breaks=c(30,50,70,90,110,130,150))+
stat_cor(aes(color = sample, label=paste
(..rr.label..,..p.label..,sep = " ~`,`~")), label.x=66, size=3.5)+
```

```
theme(axis.title.x=element_blank(), axis.text.x = element_text(size
= 12), axis.title.y = element_text(size = 14), axis.text.y =
element_text(size = 12))

svsc<-ggplot(SV, aes(x=age, y= SV_height4159))+
theme_bw()+
theme(panel.grid.major=element_blank(),
panel.grid.minor=element_blank())+
theme(legend.position="none")+
geom_point(aes(colour=sample), size=4)+
stat_smooth(aes(color = sample, fill = sample), method = "lm")+
scale_color_aaas(palette = c("default"), alpha = 1)+
scale_fill_aaas(palette = c("default"), alpha = 1)+
scale_x_continuous(expand=c(0,0), limits=c(15,85),
breaks=c(20,30,40,50,60,70,80))+
scale_y_continuous(ylab("SV (ml)/height4.159"),
limits=c(5,16),breaks=c(6,9,12,15))+
stat_cor(aes(color = sample, label=paste
(..rr.label..,..p.label..,sep = " ~`,`~")), label.x=66, size=3.5)+
theme(axis.title.x=element_blank(), axis.text.x = element_text(size
= 12), axis.title.y = element_text(size = 14), axis.text.y =
element_text(size = 12))

hr_sports <- subset(HR, group==2)
hr<-ggplot(hr_sports, aes(x=age, y= HR))+
theme_bw()+
theme(panel.grid.major=element_blank(),
panel.grid.minor=element_blank())+
theme(legend.position="none")+
geom_point(data= hr_sports, aes(colour=sample2), size=4)+
stat_smooth(data= hr_sports, aes(color = sample2, fill = sample2),
method = "lm")+
scale_color_aaas(palette = c("default"), alpha = 1)+
```

```
scale_fill_aaas(palette = c("default"), alpha = 1)+
scale_x_continuous(xlab("Age (years)"), expand=c(0,0),
limits=c(15,85), breaks=c(20,30,40,50,60,70,80))+
scale_y_continuous(ylab("HR (bpm)"),
limits=c(40,95),breaks=c(30,40,50,60,70,80,90))+
stat_cor(aes(color = sample2, label=paste
(..rr.label..,..p.label..,sep = " ~`,`~")), label.x=66, size=3.5)+
theme(axis.title.x = element_text(size = 13), axis.text.x =
element_text(size = 12), axis.title.y = element_text(size = 14),
axis.text.y = element_text(size = 12))

hrsc<-ggplot(hr_sports, aes(x=age, y= HR_height0879))+
theme_bw()+
theme(panel.grid.major=element_blank(),
panel.grid.minor=element_blank())+
theme(legend.position="none")+
geom_point(data= hr_sports, aes(colour=sample2), size=4)+
stat_smooth(data= hr_sports, aes(color = sample2, fill = sample2),
method = "lm")+
scale_color_aaas(palette = c("default"), alpha = 1)+
scale_fill_aaas(palette = c("default"), alpha = 1)+
scale_x_continuous(xlab("Age (years)"), expand=c(0,0),
limits=c(15,85), breaks=c(20,30,40,50,60,70,80))+
scale_y_continuous(ylab("HR (bpm)/weight-0.879"),
limits=c(60,160),breaks=c(70,90,110,130,150))+
stat_cor(aes(color = sample2, label=paste
(..rr.label..,..p.label..,sep = " ~`,`~")), label.x=66, size=3.5)+
theme(axis.title.x = element_text(size = 13), axis.text.x =
element_text(size = 12), axis.title.y = element_text(size = 14),
axis.text.y = element_text(size = 12))

figure2<- ggarrange(co,cosc,sv,svsc,hr,hrsc,align="hv", labels =
c("A", "D", "B","E", "C", "F"),
```

```
         ncol = 2, nrow = 3)
ggsave(file="figure2.pdf", figure2, width=14, height=10)
```

**# figure 3, CO, TEE, CO and TEE adjusted, and brain weight**

```
cols <- c("#fa5252", "#FEE61A")
figure3a.melted<-melt(figure3a,id="age")
figure3a_graf<-ggplot(figure3a.melted,
aes(x=age,y=value,fill=variable, color=variable))+
geom_point(size=4, shape=21, show.legend=T)+
scale_y_continuous(limits=c(-6.5,3.5),breaks=seq(-6,3,1))+
scale_x_continuous(limits=c(0,88), breaks=seq(0,88,5), expand =
c(0,0.5))+
theme_bw()+
theme(panel.grid.minor=element_blank(),axis.ticks =
element_blank())+
geom_hline(yintercept=0, color="grey30",size=0.5)+
scale_fill_manual(values = cols)+
scale_color_manual(values = cols)+
xlab("\nAge")+
ylab("z scores\n")+
theme(legend.position=c(0.4,0.25),
legend.text=element_text(size=15), legend.title=element_blank(),
axis.title.x = element_blank(), axis.text.x=element_text(size=14,
vjust=0.5),axis.title.y = element_text(size=16),
axis.text.y=element_text(size=14, vjust=0.5))

cols <- c("#730000","#DFC801")
figure3b.melted<-melt(figure3b,id="age")
```

```
figure3b_graf<-ggplot(figure3b.melted,
aes(x=age,y=value,fill=variable, color=variable))+
geom_point(size=4, shape=21, show.legend=T)+
scale_y_continuous(limits=c(-3.5,3.5),breaks=seq(-3,3,1))+
scale_x_continuous(limits=c(0,88), breaks=seq(0,88,5), expand =
c(0,0.5))+
theme_bw()+
theme(panel.grid.minor=element_blank(),axis.ticks =
element_blank())+
geom_hline(yintercept=0, color="grey30",size=0.5)+
scale_fill_manual(values = cols)+
scale_color_manual(values = cols)+
xlab("\nAge")+
ylab("z scores\n")+
theme(legend.position=c(0.4,0.25),
legend.text=element_text(size=15), legend.title=element_blank(),
axis.title.x = element_blank(), axis.text.x=element_text(size=14,
vjust=0.5),axis.title.y = element_text(size=16),
axis.text.y=element_text(size=14, vjust=0.5))

cols <- c("#084594","#fa5252", "#FEE61A")
figure3c.melted<-melt(figure3c,id="age")
figure3c_graf<-ggplot(figure3c.melted,
aes(x=age,y=value,fill=variable, color=variable))+
geom_point(size=4, shape=21, show.legend=T)+
scale_y_continuous(limits=c(-6.5,2.5),breaks=seq(-6,2,1))+
scale_x_continuous(limits=c(0,27), breaks=seq(0,27,2), expand =
c(0,0.5))+
theme_bw()+
theme(panel.grid.minor=element_blank(),axis.ticks =
element_blank())+
geom_hline(yintercept=0, color="grey30",size=0.5)+
scale_fill_manual(values = cols)+
```

```
scale_color_manual(values = cols)+
xlab("\nAge")+
ylab("z scores\n")+
theme(legend.position=c(0.4,0.25),
legend.text=element_text(size=15), legend.title=element_blank(),
axis.title.x = element_blank(), axis.text.x=element_text(size=14,
vjust=0.5),axis.title.y = element_text(size=16),
axis.text.y=element_text(size=14, vjust=0.5))

cols <- c("#084594","#fc3535","#DFC801", "#730000")
figure3d.melted<-melt(figure3d,id="age")
figure3d_graf<-ggplot(figure3d.melted,
aes(x=age,y=value,fill=variable, color=variable))+
geom_point(size=4, shape=21, show.legend=T)+
scale_y_continuous(limits=c(-6.5,2.5),breaks=seq(-6,2,1))+
scale_x_continuous(limits=c(0,27), breaks=seq(0,27,2), expand =
c(0,0.5))+
theme_bw()+
theme(panel.grid.minor=element_blank(),axis.ticks =
element_blank())+
geom_hline(yintercept=0, color="grey30",size=0.5)+
scale_fill_manual(values = cols)+
scale_color_manual(values = cols)+
xlab("\nAge")+
ylab("z scores\n")+
theme(legend.position=c(0.4,0.25),
legend.text=element_text(size=15),
legend.title=element_blank(),axis.title.x = element_blank(),
axis.text.x=element_text(size=14, vjust=0.5),axis.title.y =
element_blank(), axis.text.y=element_text(size=14, vjust=0.5))
```

```
figure3<-
((figure3a_graf/figure3b_graf)/(figure3c_graf|figure3d_graf))
ggsave(file="figure3.pdf", figure3, width=15, height=20)
```

# # figure 4, humans, humans ggridges, great apes

```
vh<-humans
vh1<-melt(vh,id.vars="CASE",
measure.vars=c("V8","V9","V10","V11","V12","V13","V14","V15","V16","
V17","V18","V19","V20","V21","V22","V23","V24"))
bp<-ggplot(vh1, aes(variable, value, group=CASE)) +
geom_line(size=0.5, color="#66C2A5",
show.legend=F)+theme(panel.background = element_rect(fill = "white",
colour = "black"))
bpx<-bp+theme(axis.text.x = element_text(angle = 90, hjust = 1))
bpxy<-bpx+theme(axis.text.y = element_text(angle = 90, hjust = 1))
bpxy<-bpx+theme(axis.text.y = element_text(angle = 90, hjust =
1))+scale_y_continuous(name="Asimmetry", breaks=c(-60,-40,-
20,0,20,40,60),minor_breaks=seq(-60,60,20),limits=c(-
65,65))+scale_x_discrete(name=NULL)+theme(panel.grid.major.x =
element_line(colour = "grey60"))+theme(panel.grid.major.y =
element_line(colour = "grey80"))
humansgraf<-bpxy+theme(axis.text.x=element_text(size=15,
vjust=0.5))+theme(axis.title.y=element_text(size=18),
axis.text.y=element_text(size=15,hjust=0.6))
+geom_hline(yintercept=0, color="grey30",size=1)
ggsave(file="humansgraf.pdf", humansgraf, width=16, height=8)

cols <- c("#66C2A5")
hu<-humans
hu1<-melt(hu, id.vars= c("CASE", "GROUP"), measure.vars=
c("V8","V9","V10","V11","V12","V13","V14","V15","V16","V17","V18","V
19","V20","V21","V22","V23","V24"))
```

```
hu1graf<-ggplot(hu1, aes(x=value, y=variable, fill="#66C2A5"))+
geom_density_ridges(alpha=0.75, scale=0.99,
quantile_lines=TRUE,quantile_fun=mean)+
scale_fill_manual(values = cols)+
theme(legend.position="none", panel.background = element_rect(fill =
"white", colour = "black"))+
scale_x_continuous(position="top", name=" Asimmetry", breaks=c(-60,-
40,-20,0,20,40,60),minor_breaks=seq(-60,60,20),limits=c(-65,65))+
scale_y_discrete(limits=rev, name=NULL)+theme(panel.grid.major.y =
element_line(colour = "grey60"))+theme(panel.grid.major.x =
element_line(colour = "grey80"))+
theme(axis.text.x=element_text(size=15,
vjust=0.5))+theme(axis.title.x=element_text(size=18),
axis.text.y=element_text(size=15,hjust=0.6))
+geom_vline(xintercept=0, color="grey30",size=0.8)
ggsave(file="hu1graf.pdf", hu1graf, width=8, height=16)

cols<-c("#FC8D62","#8DA0CB","#E78AC3")
vga <-greatapes
vga1<-melt(vga,id.vars=c("CASE", "GENUS"),measure.vars=
c("V8","V9","V10","V11","V12","V13","V14","V15","V16","V17","V18","V
19","V20","V21","V22","V23","V24"))
bp<-ggplot(vga1, aes(variable, value, group=CASE, color=GENUS)) +
geom_line(size=0.5, show.legend=F)+theme(panel.background =
element_rect(fill = "white", colour = "black"))+
scale_colour_manual(values=cols)
bpx<-bp+theme(axis.text.x = element_text(angle = 90, hjust = 1))
bpxy<-bpx+theme(axis.text.y = element_text(angle = 90, hjust = 1))
bpxy<-bpx+theme(axis.text.y = element_text(angle = 90, hjust =
1))+scale_y_continuous(name="Asimmetry", breaks=c(-60,-40,-
20,0,20,40,60),minor_breaks=seq(-60,60,20),limits=c(-
65,65))+scale_x_discrete(name=NULL)+theme(panel.grid.major.x =
```

```
element_line(colour = "grey60"))+theme(panel.grid.major.y =
element_line(colour = "grey80"))
greatapesgraf<- bpxy+theme(axis.text.x=element_text(size=15,
vjust=0.5))+theme(axis.title.y=element_text(size=18),
axis.text.y=element_text(size=15,hjust=0.6))
+geom_hline(yintercept=0, color="grey30",size=1)

figure4<- ggarrange(greatapesgraf, humansgraf, hu1graf,align="hv",
labels = c("A", "B", "C"),
          ncol = 3, nrow = 1)
ggsave(file="figure4.pdf", figure4, width=8, height=16)
```

# # supplementary figure 1

```
# supplementary figure 1 a: by genera

ARD1<-subset(ARD, comp_w==1)
ARD1bm<-
ARD1%>%
dabest(species, bodymass,idx =
c("Humans","Gorillas","Chimpanzees"),paired = FALSE)
ARD1bmmd<-mean_diff(ARD1bm)
graficobodymass<-plot(ARD1bmmd, palette="Set2",
rawplot.ylim=(c(40,290)), rawplot.ylabel="Body mass (kg)",
rawplot.markersize=4, rawplot.groupwidth=0.3, effsize.ylabel="Mean
difference", effsize.markersize=4, tick.fontsize=16,
axes.title.fontsize=16)

ARD2<-subset(ARD, comp_a==1)
ARD2ar<-
ARD2%>%
```

```
dabest(species, aorta,idx = c("Humans","Gorillas","Chimpanzees"),
paired = FALSE)
ARD2armd<-mean_diff(ARD2ar)
graficoaorta<-plot(ARD2armd, palette="Set2",
rawplot.ylim=(c(18,50)), rawplot.ylabel="Aortic root (mm)",
rawplot.markersize=4, rawplot.groupwidth=0.3, effsize.ylabel="Mean
difference", effsize.markersize=4, tick.fontsize=16,
axes.title.fontsize=16)

ARD3<-subset(ARD, comp_asc==1)
ARD3ars<-
ARD3%>%
dabest(species, a0236,idx = c("Humans","Gorillas","Chimpanzees"),
paired = FALSE)
ARD3arsmd<-mean_diff(ARD3ars)
graficoaortascaled<-plot(ARD3arsmd, palette="Set2",
rawplot.ylim=(c(6,16)), rawplot.ylabel="Aortic root (mm)/mass0.236",
rawplot.markersize=4, rawplot.groupwidth=0.3, effsize.ylabel="Mean
difference", effsize.markersize=4, tick.fontsize=16,
axes.title.fontsize=16)
suppfigure1_a_totalsample<-
ggarrange(graficobodymass,graficoaorta,graficoaortascaled,
align="hv", labels = c("A", "B","C"), ncol = 1, nrow = 3)

ggsave(file="suppfigure1_a_totalsample.pdf",
suppfigure1_a_totalsample, width=10, height=20)

# supplementary figure 1 b: males

ARD1m<-subset(ARD, comp_w==1 & sex=="Male")
ARD1mbm<-
ARD1m%>%
```

```
dabest(species, bodymass,idx =
c("Humans","Gorillas","Chimpanzees"),paired = FALSE)
ARD1mbmmd<-mean_diff(ARD1mbm)
graficombodymass<-plot(ARD1mbmmd, palette="Set2",
rawplot.ylim=(c(40,290)), rawplot.ylabel="Body mass (kg)",
rawplot.markersize=4, rawplot.groupwidth=0.3, effsize.ylabel="Mean
difference", effsize.markersize=4, tick.fontsize=16,
axes.title.fontsize=16)

ARD2m<-subset(ARD, comp_a==1 & sex=="Male")
ARD2mar<-
ARD2m%>%
dabest(species, aorta,idx = c("Humans","Gorillas","Chimpanzees"),
paired = FALSE)
ARD2marmd<-mean_diff(ARD2mar)
graficomaorta<-plot(ARD2marmd, palette="Set2",
rawplot.ylim=(c(18,50)), rawplot.ylabel="Aortic root (mm)",
rawplot.markersize=4, rawplot.groupwidth=0.3, effsize.ylabel="Mean
difference", effsize.markersize=4, tick.fontsize=16,
axes.title.fontsize=16)

ARD3m<-subset(ARD, comp_asc==1 & sex=="Male")
ARD3mars<-
ARD3m%>%
dabest(species, a0236,idx = c("Humans","Gorillas","Chimpanzees"),
paired = FALSE)
ARD3marsmd<-mean_diff(ARD3mars)
graficomaortascaled<-plot(ARD3marsmd, palette="Set2",
rawplot.ylim=(c(6,16)), rawplot.ylabel="Aortic root (mm)/mass0.236",
rawplot.markersize=4, rawplot.groupwidth=0.3, effsize.ylabel="Mean
difference", effsize.markersize=4, tick.fontsize=16,
axes.title.fontsize=16)
```

```
suppfigure1_b_males<-
ggarrange(graficombodymass,graficomaorta,graficomaortascaled,
align="hv", labels = c("A", "B","C"), ncol = 1, nrow = 3)

ggsave(file="suppfigure1_b_males.pdf", suppfigure1_b_males,
width=10, height=20)

# supplementary figure 1 c: females

ARD1f<-subset(ARD, comp_w==1 & sex=="Female")
ARD1fbm<-
ARD1f%>%
dabest(species, bodymass,idx =
c("Humans","Gorillas","Chimpanzees"),paired = FALSE)
ARD1fbmmd<-mean_diff(ARD1fbm)
graficofbodymass<-plot(ARD1fbmmd, palette="Set2",
rawplot.ylim=(c(40,290)), rawplot.ylabel="Body mass (kg)",
rawplot.markersize=4, rawplot.groupwidth=0.3, effsize.ylabel="Mean
difference", effsize.markersize=4, tick.fontsize=16,
axes.title.fontsize=16)

ARD2f<-subset(ARD, comp_a==1 & sex=="Female")
ARD2far<-
ARD2f%>%
dabest(species, aorta,idx = c("Humans","Gorillas","Chimpanzees"),
paired = FALSE)
ARD2farmd<-mean_diff(ARD2far)
graficofaorta<-plot(ARD2farmd, palette="Set2",
rawplot.ylim=(c(18,50)), rawplot.ylabel="Aortic root (mm)",
rawplot.markersize=4, rawplot.groupwidth=0.3, effsize.ylabel="Mean
difference", effsize.markersize=4, tick.fontsize=16,
axes.title.fontsize=16)
```

```
ARD3f<-subset(ARD, comp_asc==1 & sex=="Female")
ARD3fars<-
ARD3f%>%
dabest(species, a0236,idx = c("Humans","Gorillas","Chimpanzees"),
paired = FALSE)
ARD3farsmd<-mean_diff(ARD3fars)
graficofaortascaled<-plot(ARD3farsmd, palette="Set2",
rawplot.ylim=(c(6,16)), rawplot.ylabel="Aortic root (mm)/mass0.236",
rawplot.markersize=4, rawplot.groupwidth=0.3, effsize.ylabel="Mean
difference", effsize.markersize=4, tick.fontsize=16,
axes.title.fontsize=16)
suppfigure1_c_females<-
ggarrange(graficofbodymass,graficofaorta,graficofaortascaled,
align="hv", labels = c("A", "B","C"), ncol = 1, nrow = 3)

ggsave(file="suppfigure1_c_females.pdf", suppfigure1_c_females,
width=10, height=20)
```

# supplementary figure 2

```
# aortic root diameter, relation with other variables (to exclude
outliers from Riding, repeat using data<-subset(ARDCARDIAC,
outliers==1))

ar_sv<-ggplot(ARDCARDIAC, aes(y=aorta, x=sv))+
theme_bw()+
theme(panel.grid.major=element_blank(),
panel.grid.minor=element_blank())+
theme(legend.position="none",axis.title.y=element_blank())+
geom_point(aes(colour=sample), size=5)+
stat_smooth(method = "lm",fill="grey85", colour="black", size=0.5)+
scale_color_aaas(palette = c("default"), alpha = 1)+
```

```
scale_fill_aaas(palette = c("default"), alpha = 1)+
scale_x_continuous(xlab("\nSV (ml)"), expand=c(0,0),
limits=c(45,125), breaks=c(50,60,70,80,90,100,110,120))+
scale_y_continuous(limits=c(24,37),breaks=c(24,26,28,30,32,34,36))+
theme(axis.title.x = element_text(size = 13), axis.text.x =
element_text(size = 12), axis.text.y = element_text(size = 12))+
stat_cor(aes(label=paste (..rr.label..,..p.label..,sep = " ~`,`~")),
label.x.npc=0.1, label.y.npc=0.9,size=4)

ar_hr<-ggplot(ARDCARDIAC, aes(y=aorta, x=hr))+
theme_bw()+
theme(panel.grid.major=element_blank(),
panel.grid.minor=element_blank())+
theme(legend.position="none",axis.title.y=element_blank())+
geom_point(aes(colour=sample), size=5)+
stat_smooth(method = "lm",fill="grey85", colour="black", size=0.5)+
scale_color_aaas(palette = c("default"), alpha = 1)+
scale_fill_aaas(palette = c("default"), alpha = 1)+
scale_x_continuous(xlab("\nHR (bpm)"), expand=c(0,0),
limits=c(48,82), breaks=c(50,55,60,65,70,75,80))+
scale_y_continuous(limits=c(24,37),breaks=c(24,26,28,30,32,34,36))+
theme(axis.title.x = element_text(size = 13), axis.text.x =
element_text(size = 12), axis.text.y = element_text(size = 12))+
stat_cor(aes(label=paste (..rr.label..,..p.label..,sep = " ~`,`~")),
label.x.npc=0.1, label.y.npc=0.9,size=4)

ar_co<-ggplot(ARDCARDIAC, aes(y=aorta, x=co))+
theme_bw()+
theme(panel.grid.major=element_blank(),
panel.grid.minor=element_blank())+
theme(legend.position="none")+
geom_point(aes(colour=sample), size=5)+
stat_smooth(method = "lm",fill="grey85", colour="black", size=0.5)+
```

```
scale_color_aaas(palette = c("default"), alpha = 1)+
scale_fill_aaas(palette = c("default"), alpha = 1)+
scale_x_continuous(xlab("\nCO (L)"), expand=c(0,0),
limits=c(2.9,7.3), breaks=c(3,4,5,6,7))+
scale_y_continuous(ylab("Aortic root (mm) \n"),
limits=c(24,37),breaks=c(24,26,28,30,32,34,36))+
theme(axis.title.x = element_text(size = 13), axis.text.x =
element_text(size = 12), axis.title.y = element_text(size = 14),
axis.text.y = element_text(size = 12))+
stat_cor(aes(label=paste (..rr.label..,..p.label..,sep = " ~`,`~")),
label.x.npc=0.1, label.y.npc=0.9,size=4)

ar_lvmass<- ggplot(ARDCARDIAC, aes(y=aorta, x=lvmass))+
theme_bw()+
theme(panel.grid.major=element_blank(),
panel.grid.minor=element_blank())+
theme(legend.position="none")+
geom_point(aes(colour=sample), size=5)+
stat_smooth(method = "lm",fill="grey85", colour="black", size=0.5)+
scale_color_aaas(palette = c("default"), alpha = 1)+
scale_fill_aaas(palette = c("default"), alpha = 1)+
scale_x_continuous(xlab("\nLVMASS (g)"), expand=c(0,0),
limits=c(100,310), breaks=c(125,150,175,200,225,250,275,300))+
scale_y_continuous(ylab("Aortic root (mm) \n"),
limits=c(24,37),breaks=c(24,26,28,30,32,34,36))+
theme(axis.title.x = element_text(size = 13), axis.text.x =
element_text(size = 12), axis.text.y = element_text(size = 12))+
stat_cor(aes(label=paste (..rr.label..,..p.label..,sep = " ~`,`~")),
label.x.npc=0.1, label.y.npc=0.9,size=4)

ar_lvedd<- ggplot(ARDCARDIAC, aes(y=aorta, x=lvedd))+
theme_bw()+
```

```
theme(panel.grid.major=element_blank(),
panel.grid.minor=element_blank())+
theme(legend.position="none")+
geom_point(aes(colour=sample), size=5)+
stat_smooth(method = "lm",fill="grey85", colour="black", size=0.5)+
scale_color_aaas(palette = c("default"), alpha = 1)+
scale_fill_aaas(palette = c("default"), alpha = 1)+
scale_x_continuous(xlab("\nLVEDD (mm)"), expand=c(0,0),
limits=c(41,64), breaks=c(42,44,46,48,50,52,54,56,58,60,62))+
scale_y_continuous(ylab("Aortic root (mm) \n"),
limits=c(24,37),breaks=c(24,26,28,30,32,34,36))+
theme(axis.title.x = element_text(size = 13), axis.text.x =
element_text(size = 12), axis.title.y = element_text(size = 14),
axis.text.y = element_text(size = 12))+
stat_cor(aes(label=paste (..rr.label..,..p.label..,sep = " ~`,`~")),
label.x.npc=0.1, label.y.npc=0.9,size=4)

ar_lvesd<- ggplot(ARDCARDIAC, aes(y=aorta, x=lvesd))+
theme_bw()+
theme(panel.grid.major=element_blank(),
panel.grid.minor=element_blank())+
theme(legend.position="none",axis.title.y=element_blank())+
geom_point(aes(colour=sample), size=5)+
stat_smooth(method = "lm",fill="grey85", colour="black", size=0.5)+
scale_color_aaas(palette = c("default"), alpha = 1)+
scale_fill_aaas(palette = c("default"), alpha = 1)+
scale_x_continuous(xlab("\nLVESD (mm)"), expand=c(0,0),
limits=c(26,41), breaks=c(28,30,32,34,36,38,40))+
scale_y_continuous(ylab("Aortic root (mm) \n"),
limits=c(24,37),breaks=c(24,26,28,30,32,34,36))+
theme(axis.title.x = element_text(size = 13), axis.text.x =
element_text(size = 12), axis.text.y = element_text(size = 12))+
```

```
stat_cor(aes(label=paste (..rr.label..,..p.label..,sep = " ~`,`~")),
label.x.npc=0.1, label.y.npc=0.9,size=4)

ar_edv<- ggplot(ARDCARDIAC, aes(y=aorta, x=edv))+
theme_bw()+
theme(panel.grid.major=element_blank(),
panel.grid.minor=element_blank())+
theme(legend.position="none")+
geom_point(aes(colour=sample), size=5)+
stat_smooth(method = "lm",fill="grey85", colour="black", size=0.5)+
scale_color_aaas(palette = c("default"), alpha = 1)+
scale_fill_aaas(palette = c("default"), alpha = 1)+
scale_x_continuous(xlab("\nLVEDV (ml)"), expand=c(0,0),
limits=c(70,165), breaks=c(80,100,120,140,160))+
scale_y_continuous(ylab("Aortic root (mm) \n"),
limits=c(24,37),breaks=c(24,26,28,30,32,34,36))+
theme(axis.title.x = element_text(size = 13), axis.text.x =
element_text(size = 12), axis.title.y = element_text(size = 14),
axis.text.y = element_text(size = 12))+
stat_cor(aes(label=paste (..rr.label..,..p.label..,sep = " ~`,`~")),
label.x.npc=0.1, label.y.npc=0.9,size=4)

ar_esv<- ggplot(ARDCARDIAC, aes(y=aorta, x=esv))+
theme_bw()+
theme(panel.grid.major=element_blank(),
panel.grid.minor=element_blank())+
theme(legend.position="none",axis.title.y=element_blank())+
geom_point(aes(colour=sample), size=5)+
stat_smooth(method = "lm",fill="grey85", colour="black", size=0.5)+
scale_color_aaas(palette = c("default"), alpha = 1)+
scale_fill_aaas(palette = c("default"), alpha = 1)+
scale_x_continuous(xlab("\nLVESV (ml)"), expand=c(0,0),
limits=c(25,60), breaks=c(30,35,40,45,50,55))+
```

```
scale_y_continuous(ylab("Aortic root (mm) \n"),
limits=c(24,37),breaks=c(24,26,28,30,32,34,36))+
theme(axis.title.x = element_text(size = 13), axis.text.x =
element_text(size = 12), axis.text.y = element_text(size = 12))+
stat_cor(aes(label=paste (..rr.label..,..p.label..,sep = " ~`,`~")),
label.x.npc=0.1, label.y.npc=0.9,size=4)

#guardamos solo seis gráficos: dejamos fuera CO y HR

suppfigure2_a_total<-
ggarrange(ar_lvedd,ar_lvesd,ar_edv,ar_esv,ar_lvmass,ar_sv,align="hv"
, labels =c("A","B","C","D","E","F","G","H"),ncol = 2, nrow = 3)

ggsave(file="suppfigure2_a_total.pdf", suppfigure2_a_total,
width=10, height=15)
```

# supplementary figure 3

```
CO1 <- subset(CO, height>162.9 & age<40)
SV1 <- subset(SV, height>162.9 & age<40)
HR1 <- subset(HR, height>162.9 & age<40)

co_1<-dabest(CO1, sample,CO,idx = c("Control","Athlete"),paired =
FALSE)
a<-plot(co_1, rawplot.ylim=(c(2,10)), rawplot.ylabel="CO (L/min)",
rawplot.markersize=4, rawplot.groupwidth=0.3, effsize.ylabel="Mean
difference", effsize.markersize=4, tick.fontsize=14,
axes.title.fontsize=14)

co_2<-dabest(CO1, sample,CO_height2993,idx =
c("Control","Athlete"),paired = FALSE)
```

```
b<-plot(co_2, rawplot.ylim=(c(0.4,2)), rawplot.ylabel="CO
(L/min)/height2.993", rawplot.markersize=4, rawplot.groupwidth=0.3,
effsize.ylabel="Mean difference", effsize.markersize=4,
tick.fontsize=14, axes.title.fontsize=14)

sv_1<-dabest(SV1, sample,SV,idx = c("Control","Athlete"),paired =
FALSE)
c<-plot(sv_1, rawplot.ylim=(c(30,160)), rawplot.ylabel="SV (ml)",
rawplot.markersize=4, rawplot.groupwidth=0.3, effsize.ylabel="Mean
difference", effsize.markersize=4, tick.fontsize=14,
axes.title.fontsize=14)

sv_2<-dabest(SV1, sample,SV_height4159,idx =
c("Control","Athlete"),paired = FALSE)
d<-plot(sv_2, rawplot.ylim=(c(3,16)), rawplot.ylabel="SV
(ml)/height4.159", rawplot.markersize=4, rawplot.groupwidth=0.3,
effsize.ylabel="Mean difference", effsize.markersize=4,
tick.fontsize=14, axes.title.fontsize=14)

hr_1<-dabest(HR1, sample2,HR,idx = c("Control","Athlete"),paired =
FALSE)
e<-plot(hr_1, rawplot.ylim=(c(40,90)), rawplot.ylabel="HR (bpm)",
rawplot.markersize=4, rawplot.groupwidth=0.3, effsize.ylabel="Mean
difference", effsize.markersize=4, tick.fontsize=14,
axes.title.fontsize=14)

hr_2<-dabest(HR1, sample2,HR_height0879,idx =
c("Control","Athlete"),paired = FALSE)
f<-plot(hr_2, rawplot.ylim=(c(70,150)), rawplot.ylabel="HR
(bpm)/height-0.879", rawplot.markersize=4, rawplot.groupwidth=0.3,
effsize.ylabel="Mean difference", effsize.markersize=4,
tick.fontsize=14, axes.title.fontsize=14)
```

```
suppfigure3<- ggarrange(a,b,c,d,e,f, align="hv", labels = c("A",
"B", "C","D","E","F"),
          ncol = 2, nrow = 3)

ggsave(file=" suppfigure3.pdf", suppfigure3, width=10, height=10)
```

# supplementary figure 4

```
# organs weight (brain, heart, kidney, liver, lung, spleen)

cols <- c("#AFAEAE","#084594", "#2171B5", "#4292C6","#6BAED6",
"#9ECAE1")
organs.melted<-melt(organs,id="age")
organsgraf<-ggplot(organs.melted, aes(x=age,y=value,fill=variable,
color=variable))+
geom_point(size=2, shape=21, show.legend=T)+
scale_y_continuous(limits=c(-6.5,3.5),breaks=seq(-6,3,1))+
scale_x_continuous(limits=c(0,27), breaks=seq(0,27,2), expand =
c(0,0.5))+
theme_bw()+
theme(panel.grid.minor=element_blank(),axis.ticks =
element_blank())+
geom_hline(yintercept=0, color="grey30",size=0.5)+
scale_fill_manual(values = cols)+
scale_color_manual(values = cols)+
xlab("\nAge")+
ylab("z scores\n")+
theme(legend.title=element_blank(),legend.position=c(0.4,0.25),
legend.text=element_text(size=15), axis.title.x =
element_text(size=16), axis.text.x=element_text(size=14,
vjust=0.5),axis.title.y = element_text(size=16),
axis.text.y=element_text(size=14, vjust=0.5))
```

```
# brain weight, height and weight

cols <- c("#084594","#238B45","#005A32","#FA9FB5","#DD3497")
growthmfhwbzs.melted<-melt(growthmfhwbzs,id="age")
growthmfhwbzsgraf<-ggplot(growthmfhwbzs.melted,
aes(x=age,y=value,fill=variable, color=variable))+
geom_point(size=2, shape=21, show.legend=T)+
scale_y_continuous(limits=c(-6.5,3.5),breaks=seq(-6,3,1))+
scale_x_continuous(limits=c(0,27), breaks=seq(0,27,2), expand =
c(0,0.5))+
theme_bw()+
theme(panel.grid.minor=element_blank(),axis.ticks =
element_blank())+
geom_hline(yintercept=0, color="grey30",size=0.5)+
scale_fill_manual(values = cols)+
scale_color_manual(values = cols)+
xlab("\nAge")+
ylab("z scores\n")+
theme(legend.title=element_blank(),legend.position=c(0.4,0.25),
legend.text=element_text(size=15), axis.title.x =
element_text(size=16), axis.text.x=element_text(size=14,
vjust=0.5),axis.title.y = element_blank(),
axis.text.y=element_text(size=14, vjust=0.5))

suppfigure4<-ggarrange(organsweight, growthmfhwbzsgraf, align="hv",
labels = c("a","b"), ncol = 2, nrow = 1)
ggsave(file="suppfigure4.pdf", suppfigure4, width=20, height=10)
```

# supplementary figure 5

```
# supplementary figure 5 a: HR by sex and by athlete/control

HR_sex <-dabest(HR, sex,HR,idx = c("Male","Female"),paired = FALSE)
a<-plot(HR_sex, palette="Set2", rawplot.ylim=(c(45,85)),
rawplot.ylabel="HR (bpm)", rawplot.markersize=5,
rawplot.groupwidth=0.3, effsize.ylabel="Mean difference",
effsize.markersize=6, tick.fontsize=16, axes.title.fontsize=20)+
coord_fixed(ratio =0.75)

HR_sports <-dabest(HR, sample,HR,idx = c("Sports","Control"),paired
= FALSE)
b<-plot(HR_sports, rawplot.ylim=(c(45,85)), rawplot.ylabel="HR
(bpm)", rawplot.markersize=5, rawplot.groupwidth=0.3,
effsize.ylabel="Mean difference", effsize.markersize=6,
tick.fontsize=16, axes.title.fontsize=20)+coord_fixed(ratio =0.75)

suppfigure5<- ggarrange(a,b, align="hv", labels = c("A", "B"),ncol =
2, nrow = 1)

ggsave(file="suppfigure5.pdf", suppfigure5, width=10, height=5)
```

# supplementary figure 6

```
hr_usaperu <- subset(HR, group==1)
hr_sports <- subset(HR, group==2)

axes = theme(axis.title.x = element_text(size = 15), axis.text.x =
element_text(size = 13), axis.title.y = element_text(size = 16),
axis.text.y = element_text(size = 13))
```

```
graph<-ggplot(HR, aes(x=age, y=HR))+
theme_bw()+
theme(panel.grid.major=element_blank(),
panel.grid.minor=element_blank())+
theme(legend.position="none")+
geom_line(data= hr_usaperu, aes(colour=sample,
group=interaction(sample,percentile)),size=1.5)+
scale_colour_brewer(type = "seq", palette = "Accent", direction =1,
aesthetics = "colour") + scale_fill_brewer(type = "seq", palette =
"Accent", direction =1, aesthetics = "fill")+
scale_x_continuous(xlab("\nAge (years)"),
expand=c(0,0),limits=c(0,90), breaks=c(0,10,20,30,40,50,60,70,80))+
scale_y_continuous(ylab("HR (bpm)\n "),
limits=c(45,150),breaks=c(40,50,60,70,80,90,100,110,120,130,140,150)
)+
axes

suppfigure6<-graph+
new_scale_color()+
new_scale_fill()+
geom_point(data= hr_sports, aes(colour=sample2), size=4.5)+
stat_smooth(data= hr_sports, aes(colour = sample2, fill = sample2),
method = "lm")+
scale_color_aaas(palette = c("default"), alpha = 1)+
scale_fill_aaas(palette = c("default"), alpha = 1)

ggsave(file="suppfigure6.pdf", suppfigure6, width=20, height=10)
```
