# Supplementary material for: The aorta in humans and African great apes, and cardiac output and metabolic levels in human evolution
Source: Sci Rep. 2023 Apr 26;13:6841. doi: 10.1038/s41598-023-33675-1 (PMC10133235; doi:10.1038/s41598-023-33675-1)
Supplement: Supplementary file 8 — Supplementary Information 3. [file 41598_2023_33675_MOESM8_ESM.pdf]

References for supplementary files 2-5

1 Abergel, E. *et al.* Serial left ventricular adaptations in world-class professional cyclists - Implications for disease screening and follow-up. *Journal of the American College of Cardiology* **44**, 144-149, doi:10.1016/j.jacc.2004.02.057 (2004).

2 Abernethy, W. B., Choo, J. K. & Hutter, A. M. Echocardiographic characteristics of professional football players. *Journal of the American College of Cardiology* **41**, 280-284, doi:10.1016/s0735-1097(02)02633-5 (2003).

3 Adler, Y. *et al.* Left ventricular diastolic function in trained male weightlifters at rest and during isometric exercise. *American Journal of Cardiology* **102**, 97-101, doi:10.1016/j.amjcard.2008.02.105 (2008).

4 Agrebi, B., Tkatchuk, V., Hlila, N., Mouelhi, E. & Belhani, A. Impact of specific training and competition on myocardial structure and function in different age ranges of male handball players. *Plos One* **10**, doi:10.1371/journal.pone.0143609 (2015).

5 Akova, B., Yesilbursa, D., Sekir, U., Gur, H. & Serdar, A. Myocardial performance and aortic elastic properties in elite basketball and soccer players: Relationship with aerobic and anaerobic capacity. *Journal of Sports Science and Medicine* **4**, 185-194 (2005).

6 Andrew, C. & Hazzaa M, A. H. Echocardiographic studies in Saudi athletes. (1995).

7 Babaee Bigi, M. A. & Aslani, A. Aortic root size and prevalence of aortic regurgitation in elite strength trained athletes. *American Journal of Cardiology* **100**, 528-530, doi:10.1016/j.amjcard.2007.02.108 (2007).

8 Baggish, A. L. *et al.* Training-specific changes in cardiac structure and function: a prospective and longitudinal assessment of competitive athletes. *Journal of Applied Physiology* **104**, 1121-1128, doi:10.1152/japplphysiol.01170.2007 (2008).

9 Baggish, A. L. *et al.* The impact of endurance exercise training on left ventricular systolic mechanics. *American Journal of Physiology-Heart and Circulatory Physiology* **295**, H1109-H1116, doi:10.1152/ajpheart.00395.2008 (2008).

10 Baldi, J. C. *et al.* Left ventricular diastolic filling and systolic function of young and older trained and untrained men. *Journal of Applied Physiology* **95**, 2570-2575, doi:10.1152/japplphysiol.00441.2003 (2003).

11 Berge, H. M., Gjerdalen, G. F., Andersen, T. E., Solberg, E. E. & Steine, K. Blood pressure in professional male football players in Norway. *Journal of Hypertension* **31**, 672-679, doi:10.1097/HJH.0b013e32835eb5fe (2013).

12 Bhambhani, A. & Mathew, A. Comparison of three-dimensional echocardiography and speckle tracking echocardiography in quantification and mapping of intraventricular mechanical dyssynchrony. *Indian Heart Journal* **71**, 256-262, doi:10.1016/j.ihj.2019.04.006 (2019).

13 Bohm, P. *et al.* Right and Left Ventricular Function and Mass in Male Elite Master Athletes: A Controlled Contrast-Enhanced Cardiovascular Magnetic Resonance Study. *Circulation* **133**, 1927-1935, doi:10.1161/circulationaha.115.020975 (2016).

14 Bouvier, F., Saltin, B., Nejat, M. & Jensen-Urstad, M. Left ventricular function and perfusion in elderly endurance athletes. *Medicine and Science in Sports and Exercise* **33**, 735-740 (2001).

15 Carrick-Ranson, G. *et al.* The larger exercise stroke volume in endurance-trained men does not result from increased left ventricular early or late inflow or tissue velocities. *Acta Physiologica* **205**, 520-531, doi:10.1111/j.1748-1716.2012.02430.x (2012).

16 Cavarretta, E. *et al.* Reference values of left heart echocardiographic dimensions and mass in male peri-pubertal athletes. *European Journal of Preventive Cardiology* **25**, 1204-1215, doi:10.1177/2047487318776084 (2018).

17 Chevalier, L. *et al.* Athlete's heart patterns in elite rugby players: Effects of training specificities. *Archives of Cardiovascular Diseases* **106**, 72-78, doi:10.1016/j.acvd.2012.10.002 (2013).
18 Child, J. S., Barnard, R. J. & Taw, R. L. CARDIAC-HYPERTROPHY AND FUNCTION IN MASTER ENDURANCE RUNNERS AND SPRINTERS. *Journal of Applied Physiology* **57**, 176-181, doi:10.1152/jappl.1984.57.1.176 (1984).
19 Cottini, E. *et al.* Evaluation of left ventricular diastolic function by pulmonary venous and mitral flow velocity patterns in endurance veteran athletes. *Archives of Gerontology and Geriatrics*, 179-186, doi:10.1016/0167-4943(96)86932-9 (1996).
20 Currie, K. D. *et al.* Left Ventricular Structure and Function in Elite Swimmers and Runners. *Frontiers in Physiology* **9**, doi:10.3389/fphys.2018.01700 (2018).
21 Cuspidi, C. *et al.* Aortic root diameter and risk of cardiovascular events in a general population: data from the PAMELA study. *Journal of Hypertension* **32**, 1879-1887, doi:10.1097/hjh.0000000000000264 (2014).
22 Cuspidi, C. *et al.* Aortic root dilatation in hypertensive patients: A multicenter survey in echocardiographic practice. *Blood Pressure* **20**, 267-273, doi:10.3109/08037051.2011.565556 (2011).
23 Daimon, M. *et al.* Normal Values of Echocardiographic Parameters in Relation to Age in a Healthy Japanese Population-The JAMP Study. *Circulation Journal* **72**, 1859-1866, doi:10.1253/circj.CJ-08-0171 (2008).
24 Davis, C. K., Dyar, D. A., Vargas, L. A. & Grossfeld, P. D. Cardiovascular and Musculoskeletal Assessment of Elite US Volleyball Players. *Clinical Journal of Sport Medicine* **25**, 546-550, doi:10.1097/jsm.0000000000000178 (2015).
25 Degens, H., Stasiulis, A., Skurvydas, A., Statkeviciene, B. & Venckunas, T. Physiological comparison between non-athletes, endurance, power and team athletes. *European Journal of Applied Physiology* **119**, 1377-1386, doi:10.1007/s00421-019-04128-3 (2019).
26 Dibello, V. *et al.* LEFT-VENTRICULAR PERFORMANCE AND ULTRASONIC MYOCARDIAL QUANTITATIVE REFLECTIVITY IN ENDURANCE SENIOR ATHLETES - AN ECHOCARDIOGRAPHIC STUDY. *European Heart Journal* **14**, 358-363, doi:10.1093/eurheartj/14.3.358 (1993).
27 Donal, E. *et al.* Comparison of the Heart Function Adaptation in Trained and Sedentary Men After 50 and Before 35 Years of Age. *American Journal of Cardiology* **108**, 1029-1037, doi:10.1016/j.amjcard.2011.05.043 (2011).
28 Doronina, A. *et al.* The Female Athlete's Heart: Comparison of Cardiac Changes Induced by Different Types of Exercise Training Using 3D Echocardiography. *Biomed Research International* **2018**, doi:10.1155/2018/3561962 (2018).
29 Dzudie, A. *et al.* Ultrasonographic study of left ventricular function at rest in a group of highly trained black African handball players. *European Journal of Echocardiography* **8**, 122-127, doi:10.1016/j.euje.2006.02.006 (2007).
30 Edenfield, K. M. *et al.* Echocardiographic measurements of left ventricular end-diastolic diameter and interventricular septal diameter in collegiate football athletes at preparticipation evaluation referenced to body surface area. *Bmj Open Sport & Exercise Medicine* **5**, doi:10.1136/bmjsem-2018-000488 (2019).
31 Engel, D. J., Schwartz, A. & Homma, S. Athletic Cardiac Remodeling in US Professional Basketball Players. *Jama Cardiology* **1**, 80-87, doi:10.1001/jamacardio.2015.0252 (2016).
32 Fisman, E. Z. *et al.* Comparison of left ventricular function using isometric exercise Doppler echocardiography in competitive runners and weightlifters verses sedentary individuals. *American Journal of Cardiology* **79**, 355-359, doi:10.1016/s0002-9149(96)00761-8 (1997).
33 Fisman, E. Z. *et al.* Effect of intensive resistance training on isotonic exercise Doppler indexes of left ventricular systolic function. *American Journal of Cardiology* **89**, 887-+, doi:10.1016/s0002-9149(02)02210-5 (2002).

34 Fleg, J. L. *et al.* LEFT-VENTRICULAR DIASTOLIC FILLING PERFORMANCE IN OLDER MALE-ATHLETES. *Jama-Journal of the American Medical Association* **273**, 1371-1375, doi:10.1001/jama.273.17.1371 (1995).
35 Fukuda, S. *et al.* Normal Values of Real-Time 3-Dimensional Echocarcliographic Parameters in a Healthy Japanese Population - The JAMP-3D Study. *Circulation Journal* **76**, 1177-1181, doi:10.1253/circj.CJ-11-1256 (2012).
36 Galanti, G., Stefani, L., Mascherini, G., Di Tante, V. & Toncelli, L. Left ventricular remodeling and the athlete's heart, irrespective of quality load training. *Cardiovascular Ultrasound* **14**, doi:10.1186/s12947-016-0088-x (2016).
37 Galetta, F. *et al.* Left ventricular diastolic function and carotid artery wall in elderly athletes and sedentary controls. *Biomedicine & Pharmacotherapy* **58**, 437-442, doi:10.1016/j.biopha.2004.08.008 (2004).
38 Galetta, F. *et al.* QT dispersion in elderly athletes with left ventricular hypertrophy. *International Journal of Sports Medicine* **24**, 233-237, doi:10.1055/s-2003-39499 (2003).
39 Gates, P. E., Tanaka, H., Graves, J. & Seals, D. R. Left ventricular structure and diastolic function with human ageing - Relation to habitual exercise and arterial stiffness. *European Heart Journal* **24**, 2213-2220, doi:10.1016/j.ehj.2003.09.026 (2003).
40 George, K. P., Gates, P. E., Whyte, G., Fenoglio, R. A. & Lea, R. Echocardiographic examination of cardiac structure and function in elite cross trained male and female Alpine skiers. *British Journal of Sports Medicine* **33**, 93-98, doi:10.1136/bjsm.33.2.93 (1999).
41 Giada, F. *et al.* Cardiovascular adaptations to endurance training and detraining in young and older athletes. *International Journal of Cardiology* **65**, 149-155, doi:10.1016/s0167-5273(98)00102-8 (1998).
42 Grace, F. *et al.* High intensity interval training (HIIT) improves resting blood pressure, metabolic (MET) capacity and heart rate reserve without compromising cardiac function in sedentary aging men. *Experimental Gerontology* **109**, 75-81, doi:10.1016/j.exger.2017.05.010 (2018).
43 Grossman, A., Benderly, M., Prokupetz, A., Gordon, B. & Kalter-Leibovici, O. M-mode echocardiographic values in a cohort of young healthy individuals. *Journal of Cardiovascular Medicine* **16**, 45-50, doi:10.2459/JCM.0b013e3283641bf0 (2015).
44 Gyimes, Z., Pavlik, G. & Simor, T. Magnetic Resonance Imaging study for the comparative measurement of cardiac parameters between endurance and power and fast-power athletes. *Journal of Clinical and Basic Cardiology* **7**, 15-18 (2004).
45 Hasdemir, H. *et al.* Aortic properties and atrial electrophysiology in the young and old football players. *Revista Da Associacao Medica Brasileira* **57**, 276-281 (2011).
46 Heron, N. & Cupples, M. The health profile of football/soccer players in Northern Ireland - a review of the uefa pre-participation medical screening procedure. *BMC sports science, medicine & rehabilitation* **6**, 5-5, doi:10.1186/2052-1847-6-5 (2014).
47 Hosseini, K., Mazaheri, R., Khoddami Vishteh, H., Mansournia, M. & Angoorani, H. Cardiac Function and Morphological Adaptations in Endurance and Resistance Athletes: Evaluation using a new Method. *World academi of science, engineering and Technology* **59**, 366-369 (2011).
48 Johnson, C. *et al.* Cardiac structure and function in elite Native Hawaiian and Pacific Islander Rugby Football League athletes: an exploratory study. *International Journal of Cardiovascular Imaging* **34**, 725-734, doi:10.1007/s10554-017-1285-x (2018).
49 Jungblut, P. R. *et al.* Echocardiographic Doppler evaluation of left ventricular diastolic filling in older, highly trained male endurance athletes. *Echocardiography-a Journal of Cardiovascular Ultrasound and Allied Techniques* **17**, 7-16, doi:10.1111/j.1540-8175.2000.tb00988.x (2000).
50 Kay, S. *et al.* Rugby Player's Aorta: Alarming Prevalence of Ascending Aortic Dilatation and Effacement in Elite Rugby Players. *Heart Lung and Circulation* **29**, 196-201, doi:10.1016/j.hlc.2019.06.714 (2020).

51 Kervio, G. *et al.* Alterations in echocardiographic and electrocardiographic features in Japanese professional soccer players: comparison to African-Caucasian ethnicities. *European Journal of Preventive Cardiology* **20**, 880-888, doi:10.1177/2047487312447905 (2013).
52 Kim, J. H. *et al.* Impact of American-Style Football Participation on Vascular Function. *American Journal of Cardiology* **115**, 262-267, doi:10.1016/j.amjcard.2014.10.033 (2015).
53 Kneffel, Z. *et al.* Relationship between relative aerobic power and echocardiographic characteristics in male athletes. *Echocardiography-a Journal of Cardiovascular Ultrasound and Allied Techniques* **24**, 901-910, doi:10.1111/j.1540-8175.2007.00494.x (2007).
54 Kou, S. *et al.* Echocardiographic reference ranges for normal cardiac chamber size: results from the NORRE study. *European Heart Journal-Cardiovascular Imaging* **15**, 680-690, doi:10.1093/ehjci/jet284 (2014).
55 Kozakova, M. *et al.* Coronary vasodilator capacity and epicardial vessel remodeling in physiological and hypertensive hypertrophy. *Hypertension* **36**, 343-349, doi:10.1161/01.Hyp.36.3.343 (2000).
56 Lalande, S. & Baldi, J. C. Left ventricular mass in elite olympic weight lifters. *American Journal of Cardiology* **100**, 1177-1180, doi:10.1016/j.amjcard.2007.05.036 (2007).
57 Lee, L. S., Mariani, J. A., Sasson, Z. & Goodman, J. M. Exercise with a Twist: Left Ventricular Twist and Recoil in Healthy Young and Middle-Aged Men, and Middle-Aged Endurance-Trained Men. *Journal of the American Society of Echocardiography* **25**, 986-993, doi:10.1016/j.echo.2012.05.018 (2012).
58 Leung, N. K. Echocardiographic values for cardiac dimensions and left ventricular mass of normal Chinese adults: a pilot study. *Journal of Diagnostic Medical Sonography* **25**, 300-309 (2009).
59 MacFarlane, N., Northridge, D. B., Wright, A. R., Grant, S. & Dargie, H. J. A comparative study of left ventricular structure and function in elite athletes. *British journal of sports medicine* **25**, 45-48 (1991).
60 Marek, J. *et al.* Normative reference ranges for echocardiographic chamber dimensions in a healthy Central European population: results from the Czech post-MONICA survey. *Cardiovascular Ultrasound* **17**, doi:10.1186/s12947-019-0172-0 (2019).
61 Matelot, D. *et al.* Cardiovascular benefits of endurance training in seniors: 40 is not too late to start. *International journal of sports medicine* **37**, 625-632 (2016).
62 Maufrais, C. *et al.* Left ventricles of aging athletes: better untwisters but not more relaxed during exercise. *Clinical Research in Cardiology* **106**, 884-892 (2017).
63 Maufrais, C. *et al.* Endurance Training Minimizes Age-Related Changes of Left Ventricular Twist-Untwist Mechanics. *Journal of the American Society of Echocardiography* **27**, 1208-1215, doi:10.1016/j.echo.2014.07.007 (2014).
64 Mejia, C. R. *et al.* Values of heart rate at rest in children and adults living at different altitudes in the Andes. *Plos One* **14**, doi:10.1371/journal.pone.0213014 (2019).
65 Miki, T., Yokota, Y., Seo, T. & Yokoyama, M. ECHOCARDIOGRAPHIC FINDINGS IN 104 PROFESSIONAL CYCLISTS WITH FOLLOW-UP-STUDY. *American Heart Journal* **127**, 898-905, doi:10.1016/0002-8703(94)90559-2 (1994).
66 Milliken, M. C., Stray-Gundersen, J., Peshock, R. M., Katz, J. & Mitchell, J. H. Left ventricular mass as determined by magnetic resonance imaging in male endurance athletes. *The American journal of cardiology* **62**, 301-305 (1988).
67 Molmen, H. E., Wisloff, U., Aamot, I. L., Stoylen, A. & Ingul, C. B. Aerobic interval training compensates age related decline in cardiac function. *Scandinavian Cardiovascular Journal* **46**, 163-171, doi:10.3109/14017431.2012.660192 (2012).
68 Moro, A. S., Okoshi, M. P., Padovani, C. R. & Okoshi, K. Doppler echocardiography in athletes from different sports. *Medical Science Monitor* **19**, 187-193 (2013).
69 Nishimura, T., Yamada, Y. & Kawai, C. ECHOCARDIOGRAPHIC EVALUATION OF LONG-TERM EFFECTS OF EXERCISE ON LEFT-VENTRICULAR HYPERTROPHY AND FUNCTION IN PROFESSIONAL BICYCLISTS. *Circulation* **61**, 832-840, doi:10.1161/01.Cir.61.4.832 (1980).

70 Northcote, R. J., McKillop, G., Todd, I. C. & Canning, G. P. THE EFFECT OF HABITUAL SUSTAINED ENDURANCE EXERCISE ON CARDIAC STRUCTURE AND FUNCTION. *European Heart Journal* **11**, 17-22, doi:10.1093/oxfordjournals.eurheartj.a059585 (1990).
71 Nottin, S., Nguyen, L. D., Terbah, M. & Obert, P. Long-term endurance training does not prevent the age-related decrease in left ventricular relaxation properties. *Acta physiologica scandinavica* **181**, 209-215 (2004).
72 Nwabuo, C. C. *et al.* Association of Aortic Root Dilation from Early Adulthood to Middle Age with Cardiac Structure and Function: The CARDIA Study. *Journal of the American Society of Echocardiography* **30**, 1172-1179, doi:10.1016/j.echo.2017.08.001 (2017).
73 Oates, S. A. *et al.* Scaling to produce size-independent indices of echocardiographic derived aortic root dimensions in elite Rugby Football League players. *Ultrasound* **27**, 94-100, doi:10.1177/1742271x18818607 (2019).
74 Olsen, R. H. *et al.* Age-related decline in mitral peak diastolic velocities is unaffected in well-trained runners. *Scandinavian Cardiovascular Journal* **49**, 183-192, doi:10.3109/14017431.2015.1049654 (2015).
75 Ostchega, Y., Porter, K. S., Hughes, J., Dillon, C. F. & Nwankwo, T. Resting pulse rate reference data for children, adolescents, and adults: United States, 1999-2008. *National health statistics reports*, 1-16 (2011).
76 Otsuki, T. *et al.* Relationship between arterial stiffness and athletic training programs in young adult men. *American Journal of Hypertension* **20**, 967-973, doi:10.1016/j.amjhyper.2007.05.001 (2007).
77 Oxborough, D. *et al.* Left and right ventricular longitudinal strain-volume/area relationships in elite athletes. *International Journal of Cardiovascular Imaging* **32**, 1199-1211, doi:10.1007/s10554-016-0910-4 (2016).
78 Pavlik, G. *et al.* Echocardiographic data in Hungarian top-level water polo players. *Medicine and Science in Sports and Exercise* **37**, 323-328, doi:10.1249/01.Mss.0000152805.34215.97 (2005).
79 Pelliccia, A. *et al.* Prevalence and Clinical Significance of Aortic Root Dilation in Highly Trained Competitive Athletes. *Circulation* **122**, 698-U621, doi:10.1161/circulationaha.109.901074 (2010).
80 Prajapati, D., Sharma, D., Baidya, S., Shakya, U. & Shrestha, N. Normal echocardiographic parameters of healthy adult individuals working in National Heart Centre. *Nepalese Heart Journal* **9**, 3-6 (2012).
81 Prakken, N. H. J. *et al.* Head-to-head comparison between echocardiography and cardiac MRI in the evaluation of the athlete's heart. *British Journal of Sports Medicine* **46**, 348-354, doi:10.1136/bjsm.2010.077669 (2012).
82 Reed, C. M., Richey, P. A., Pulliam, D. A., Somes, G. W. & Alpert, B. S. AORTIC DIMENSIONS IN TALL MEN AND WOMEN. *American Journal of Cardiology* **71**, 608-610, doi:10.1016/0002-9149(93)90523-f (1993).
83 Reifsteck, F. *et al.* Echocardiographic measurements of aortic root diameter (ARD) in collegiate football Athletes at pre-participation evaluation. *Bmj Open Sport & Exercise Medicine* **5**, doi:10.1136/bmjsem-2019-000546 (2019).
84 Riding, N. R. *et al.* Do big athletes have big hearts? Impact of extreme anthropometry upon cardiac hypertrophy in professional male athletes. *British Journal of Sports Medicine* **46**, 90-97, doi:10.1136/bjsports-2012-091258 (2012).
85 Rileyhagan, M. *et al.* LEFT-VENTRICULAR DIMENSIONS AND MASS USING MAGNETIC-RESONANCE-IMAGING IN FEMALE ENDURANCE ATHLETES. *American Journal of Cardiology* **69**, 1067-1074, doi:10.1016/0002-9149(92)90865-v (1992).
86 Sagiv, M., Goldhammer, E., Ben-Sira, D. & Amir, R. What maintains energy supply at peak aerobic exercise in trained and untrained older men? *Gerontology* **53**, 357-361, doi:10.1159/000104898 (2007).

87 Santoro, A. *et al.* Left Ventricular Twisting Modifications in Patients with Left Ventricular Concentric Hypertrophy at Increasing After-Load Conditions. *Echocardiography-a Journal of Cardiovascular Ultrasound and Allied Techniques* **31**, 1265-1273, doi:10.1111/echo.12555 (2014).
88 Saura, D. *et al.* Two-dimensional transthoracic echocardiographic normal reference ranges for proximal aorta dimensions: results from the EACVI NORRE study. *European Heart Journal-Cardiovascular Imaging* **18**, 167-179, doi:10.1093/ehjci/jew053 (2017).
89 Scharhag, J. *et al.* Athlete's heart - Right and left ventricular mass and function in male endurance athletes and untrained individuals determined by magnetic resonance imaging. *Journal of the American College of Cardiology* **40**, 1856-1863, doi:10.1016/s0735-1097(02)02478-6 (2002).
90 Schmidt, J. F. *et al.* Cardiovascular function is better in veteran football players than age-matched untrained elderly healthy men. *Scandinavian Journal of Medicine & Science in Sports* **25**, 61-69, doi:10.1111/sms.12153 (2015).
91 Schvartzman, P. R. *et al.* Normal values of echocardiographic measurements. A population-based study. *Arquivos brasileiros de cardiologia* **75**, 107-114 (2000).
92 Seals, D. R. *et al.* ENHANCED LEFT-VENTRICULAR PERFORMANCE IN ENDURANCE-TRAINED OLDER MEN. *Circulation* **89**, 198-205, doi:10.1161/01.Cir.89.1.198 (1994).
93 Shave, R. E. *et al.* Selection of endurance capabilities and the trade-off between pressure and volume in the evolution of the human heart. *Proceedings of the National Academy of Sciences of the United States of America* **116**, 19905-19910, doi:10.1073/pnas.1906902116 (2019).
94 Silva, D. V. *et al.* Comparison of Cardiac and Vascular Parameters in Powerlifters and Long-Distance Runners: Comparative Cross-Sectional Study. *Arquivos Brasileiros De Cardiologia* **111**, 772-781, doi:10.5935/abc.20180167 (2018).
95 Sorimachi, H. *et al.* Sex differences in left ventricular afterload and diastolic function are independent from the aortic size. *Plos One* **14**, doi:10.1371/journal.pone.0214907 (2019).
96 Sotiriou, P. *et al.* Arterial adaptations in athletes of dynamic and static sports disciplines - a pilot study. *Clinical Physiology and Functional Imaging* **39**, 183-191, doi:10.1111/cpf.12554 (2019).
97 Spence, A. L. *et al.* A prospective randomised longitudinal MRI study of left ventricular adaptation to endurance and resistance exercise training in humans. *Journal of Physiology-London* **589**, 5443-5452, doi:10.1113/jphysiol.2011.217125 (2011).
98 Steding, K. *et al.* Relation between cardiac dimensions and peak oxygen uptake. *Journal of Cardiovascular Magnetic Resonance* **12**, doi:10.1186/1532-429x-12-8 (2010).
99 Sun, B., Ma, J. Z., Yong, Y. H. & Yuan, Y. L. V. The upper limit of physiological cardiac hypertrophy in elite male and female athletes in China. *European Journal of Applied Physiology* **101**, 457-463, doi:10.1007/s00421-007-0517-5 (2007).
100 Szauder, I., Kovacs, A. & Pavlik, G. Comparison of left ventricular mechanics in runners versus bodybuilders using speckle tracking echocardiography. *Cardiovascular Ultrasound* **13**, doi:10.1186/s12947-015-0002-y (2015).
101 Tumuklu, M. M., Etikan, I. & Cinar, C. S. Left ventricular function in professional football players evaluated by tissue Doppler imaging and strain imaging. *International Journal of Cardiovascular Imaging* **24**, 25-35, doi:10.1007/s10554-007-9218-8 (2008).
102 Uberoi, A., Sadik, J., Lipinski, M. J., Van Le, V. & Froelicher, V. Association Between Cardiac Dimensions and Athlete Lineup Position: Analysis Using Echocardiography in NCAA Football Team Players. *Physician and Sportsmedicine* **41**, 58-66, doi:10.3810/psm.2013.09.2025 (2013).
103 van Grootel, R. W. J., Menting, M. E., McGhie, J., Roos-Hesselink, J. W. & van den Bosch, A. E. Echocardiographic chamber quantification in a healthy Dutch population. *Netherlands Heart Journal* **25**, 682-690, doi:10.1007/s12471-017-1035-7 (2017).

104 Vasconcellos, H. D. *et al.* Cumulative blood pressure from early adulthood to middle age is associated with left atrial remodelling and subclinical dysfunction assessed by three-dimensional echocardiography: a prospective post hoc analysis from the coronary artery risk development in young adults study. *European Heart Journal-Cardiovascular Imaging* **19**, 977-984, doi:10.1093/ehjci/jey086 (2018).
105 Vasiliauskas, D., Venckunas, T., Marcinkeviciene, J. & Bartkeviciene, A. Development of structural cardiac adaptation in basketball players. *European Journal of Cardiovascular Prevention & Rehabilitation* **13**, 985-989, doi:10.1097/01.hjr.0000238394.04600.fc (2006).
106 Vinereanu, D. *et al.* Left ventricular long-axis diastolic function is augmented in the hearts of endurance-trained compared with strength-trained athletes. *Clinical Science* **103**, 249-257, doi:10.1042/cs1030249 (2002).
107 Vriz, O. *et al.* Normal Values of Aortic Root Dimensions in Healthy Adults. *American Journal of Cardiology* **114**, 921-927, doi:10.1016/j.amjcard.2014.06.028 (2014).
108 Weiner, R. B. *et al.* Blood Pressure and Left Ventricular Hypertrophy During American-Style Football Participation. *Circulation* **128**, 524-531, doi:10.1161/circulationaha.113.003522 (2013).
109 Zeppilli, P. *et al.* ECHOCARDIOGRAPHIC SIZE OF CONDUCTANCE VESSELS IN ATHLETES AND SEDENTARY PEOPLE. *International Journal of Sports Medicine* **16**, 38-44, doi:10.1055/s-2007-972961 (1995).
110 Zumbakyte-Sermuksniene, R. *et al.* Exploring the Aortic Root Diameter and Left Ventricle Size among Lithuanian Athletes. *Medicina-Lithuania* **55**, doi:10.3390/medicina55060271 (2019).
